# An angiogenesis-associated gene-based signature predicting prognosis and immunotherapy efficacy of head and neck squamous cell carcinoma patients

**DOI:** 10.1007/s00432-024-05606-8

**Published:** 2024-02-12

**Authors:** Bangjie Chen, Yanxun Han, Shuyan Sheng, Jianyi Deng, Emely Vasquez, Vicky Yau, Muzi Meng, Chenyu Sun, Tao Wang, Yu Wang, Mengfei Sheng, Tiangang Wu, Xinyi Wang, Yuchen Liu, Ning Lin, Lei Zhang, Wei Shao

**Affiliations:** 1 College & Hospital of Stomatology, Key Lab. of Oral Diseases Research of Anhui Province, Anhui Medical University, Hefei, China; 2 The First Affiliated Hospital (First Clinical Medical College), Anhui Medical University, Hefei, China; 3 CUNY School of Medicine, New York, USA; 4 Division of Oral and Maxillofacial Surgery, NewYork Presbyterian (Columbia Irving Medical Center), New York, USA; 5 UK Program Site, American University of the Caribbean School of Medicine, Preston, UK; 6 Bronxcare Health System, New York, USA; 7 The Second Affiliated Hospital of Anhui Medical University, Hefei, China; 8 The Affiliated Chuzhou Hospital of Anhui Medical University, The First People’s Hospital of Chuzhou, Chuzhou, China; 9 Department of Microbiology and Parasitology (Anhui Provincial Laboratory of Pathogen Biology), School of Basic Medical Sciences, Anhui Medical University, Hefei, China

**Keywords:** Angiogenesis-associated gene, HNSCC, Diagnosis, Prognostic signature, Immunotherapy

## Abstract

**Objectives:**

To develop a model that can assist in the diagnosis and prediction of prognosis for head and neck squamous cell carcinoma (HNSCC).

**Materials and methods:**

Data from TCGA and GEO databases were used to generate normalized gene expression data. Consensus Cluster Plus was used for cluster analysis and the relationship between angiogenesis-associated gene (AAG) expression patterns, clinical characteristics and survival was examined. Support vector machine (SVM) and least absolute shrinkage and selection operator (LASSO) analyzes and multiple logistic regression analyzes were performed to determine the diagnostic model, and a prognostic nomogram was constructed using univariate and multivariate Cox regression analyses. ESTIMATE, XCELL, TIMER, QUANTISEQ, MCPCOUNTER, EPIC, CIBERSORT-ABS, CIBERSORT algorithms were used to assess the immune microenvironment of HNSCC patients. In addition, gene set enrichment analysis, treatment sensitivity analysis, and AAGs mutation studies were performed. Finally, we also performed immunohistochemistry (IHC) staining in the tissue samples.

**Results:**

We classified HNSCC patients into subtypes based on differences in AAG expression from TCGA and GEO databases. There are differences in clinical features, TME, and immune-related gene expression between two subgroups. We constructed a HNSCC diagnostic model based on nine AAGs, which has good sensitivity and specificity. After further screening, we constructed a prognostic risk signature for HNSCC based on six AAGs. The constructed risk score had a good independent prognostic significance, and it was further constructed into a prognostic nomogram together with age and stage. Different prognostic risk groups have differences in immune microenvironment, drug sensitivity, gene enrichment and gene mutation.

**Conclusion:**

We have constructed a diagnostic and prognostic model for HNSCC based on AAG, which has good performance. The constructed prognostic risk score is closely related to tumor immune microenvironment and immunotherapy response.

**Supplementary Information:**

The online version contains supplementary material available at 10.1007/s00432-024-05606-8.

## Introduction

Head and neck squamous cell carcinoma (HNSCC) is the most frequent malignant tumor of head and neck region with the sixth-highest incidence worldwide (Johnson et al. 2020; Ferlay et al. 2019).The frequency of HNSCC is increasing, and GLOBOCAN expects a 30% increase in HNSCC patients by 2030 (Johnson et al. 2020). Traditionally the diagnosis of HNSCC relies on biopsy. Surgery, radiation, and chemotherapy remain the cornerstones of contemporary treatment for HNSCC. However, more than 65% of previously treated HNSCC patients will experience local recurrence or distant metastasis (Kok 2020; Chow 2020). Most of the patients with recurrent and metastatic HNSCC cannot be treated with radical treatment, whose overall survival rate is low (Chow 2020; Cramer et al. 2019). After FDA first authorized immune checkpoint inhibitors (ICIs) to be used as second-line treatment for metastatic or incurable HNSCC in 2016, they promptly became the first-line treatment by 2019 (Cramer et al. 2019). However, only a subset of HNSCC patients have responded to ICIs so far, and certain HNSCC populations seem to be notably resistant (Farlow et al. 2021). Therefore, to aid in the accuracy and optimization of HNSCC therapy, it is required to categorize patients with various features into several subtypes and find efficient diagnostic and prognostic biomarkers.

Angiogenesis is a key step in tumor growth and metastasis (Hanahan and Weinberg 2011). Vascular endothelial growth factor (VEGF), which is directly associated with patient prognosis, is an abundantly expressed angiogenic factor in HNSCC (Vassilakopoulou et al. 2015). Therefore, an appealing therapeutic approach for the treatment of HNSCC patients involves blocking angiogenesis, particularly the VEGF pathway (Prusch 1986). However, monotherapy of anti-angiogenic drugs usually exhibits lower efficacy. Adding anti-angiogenic agents to chemotherapy or other targeted agents is a promising therapeutic option (Vassilakopoulou et al. 2015). Currently, the majority of research focuses on how a specific angiogenic gene affects HNSCC (Butkiewicz et al. 2020; Siemert et al. 2021). Therefore, it is critical to comprehensively study the HNSCC classification based on multiple angiogenic factors.

In recent years, immunotherapy has emerged, and its effectiveness and advantages have been confirmed in a variety of tumor clinical studies, providing a new alternative for cancer treatment (Chalabi et al. 2020; Yang 2015; McArthur and Page 2016). Common immunotherapy strategies include chimeric antigen receptor (CAR) T cell therapy, tumor vaccines, and ICIs and nonspecific immunomodulators (Qing et al. 2022; Jahanafrooz et al. 2020). Among them, ICI research on programmed cell death protein 1 (PD-1), programmed death ligand 1 (PD-L1), and cytotoxic T lymphocyte antigen 4 (CTLA-4) is expanding, and clinical data have also attested to the drugs’ efficacy and safety (Jahanafrooz et al. 2020; Al-Mterin et al. 2022; Saleh et al. 2020; O'Donnell et al. 2019).However, immunotherapy is only beneficial in certain patient groups(Hegde and Chen 2020; Lesch and Gill 2021). There is mounting evidence supporting that the tumor microenvironment (TME) influences the tumor response to immunotherapy (Bader et al. 2020; Petitprez et al. 2020).

The surrounding environment of tumor cell development, proliferation, and metastasis is referred to as the TME, which is a complex and comprehensive system (Bejarano et al. 2021; Quail and Joyce 2013). In the TME, tumor cells can achieve immune escape by reducing immunogenicity, inducing changes in immunosuppression related cells and molecules (O’Donnell et al. 2019; Lei et al. 2020; Walsh et al. 2019). It is worth noting that neovascularization is a prominent feature of TME, which contributes to tumor growth and metastasis (Hanahan and Weinberg 2011). Usually, neovascularization provides nutrition for primary tumors that are constantly growing and infiltrating. In response, as they expand, tumor cells also emit a range of chemicals that hasten the creation of new capillaries inside the tumor (Ramjiawan et al. 2017; Wang et al. 2015). In addition, tumor cells actively encourage angiogenesis and inflammation to avoid immune system detection and removal (Dufies et al. 2019; Hanahan and Coussens 2012). Therefore, understanding the intricate connection between angiogenesis and TME helps to distinguish various tumor immune subtypes and enhances the ability to predict immunotherapy outcomes.

In this research, we subtyped HNSCC patients based on expression differences of angiogenesis-associated genes (AAGs) from TCGA and GEO databases, and systematically assessed the relationship between clinical features, TME, and immune-related gene expression across clusters. In addition, we also constructed diagnostic and prognostic models. The effectiveness of immunotherapy in HNSCC patients could be predicted by prognostic models. Moreover, we also conducted medication sensitivity tests on HNSCC patients in various prognostic risk categories. In summary, our research may contribute to the diagnosis, prognosis prediction, and treatment planning of HNSCC. Finally, we also performed immunohistochemistry (IHC) staining, western blotting and RT-qPCR in tissue samples to verify the expression changes of related AGG genes in HNSCC.

## Materials and methods

### Data collection and processing

The TCGA database, data sets GSE41613 (Han et al. 2022; Liu et al. 2023) and GSE127165 (Yan et al. 2022) from the GEO database, and the clinical parameters of the HNSCC samples were downloaded and used to generate the normalized gene expression data. In addition, the combat function is used to eliminate batch effect differences between datasets. Among them, sample from TCGA and GSE41613 were used to construct the prognostic signature, while samples from TCGA database and GSE127165 dataset were used to construct the diagnostic model. Samples that lacked critical clinicopathological or survival data were eliminated from further examination. From MSigDB Team, 35 different types of AAGs were acquired.

### Clustering based on AAGs and analysis of their relationship with HNSCC clinical characteristics, survival and biological function

“Consensus Cluster Plus” was used to subgroup the tumor sample into several clusters. After that, principal component analysis (PCA) is used to confirm the accuracy of clustering. Todetermine the clinical significance of classified clusters, the relationships among AAG expression patterns, clinical variables and survival results were examined. Clinical variables include age, gender, stage and survival state. The overall survival (OS) differences between various categorization clusters were assessed using the Kaplan–Meier analysis technique. In addition, KEGG and GO gene sets were used for gene set variation analysis (GSVA).

### Analysis of HNSCC immune microenvironment

The ESTIMATE algorithm was used to assess the Immune and Stromal score of HNSCC patients, and the CIBERSORT algorithm was used to determine the level of 22 immune cell subtypes in each patient, to investigate the differences of immune microenvironment and immune-related gene expression between different subtypes of HNSCC. The ssGSEA algorithm detected the infiltrated fraction of immune cells. Differences in human leukocyte antigen (HLA) and immune checkpoint expression between subtypes were also assessed. The Wilcoxon symbolic-rank test was performed to examine the variation in immune infiltration cell composition between the high-risk and low-risk groups.

### Construction and validation of HNSCC diagnostic model and prognostic signature

Support vector machine (SVM) analysis based on the “e1071” R package and least absolute shrinkage and selection operator (LASSO) analysis were used to evaluate the intersection genes before building a diagnostic model using multiple logistic regression analysis. It is worth noting that LASSO, ElasticNet, and Net are all regularization based computational methods. ElasticNet and Net typically exhibit less overfitting and better predictive performance compared to LASSO (Pak et al. 2020). However, due to the simultaneous introduction of L1 and L2 regularization, selecting appropriate regularization parameters becomes a more complex step. Therefore, LASSO and Cox proportional hazards regression have become more widely used model building methods, especially in clinical medical research. To verify the effectiveness of the diagnostic model, receiver operating characteristic (ROC) curves were utilized.

We used univariate Cox proportional hazards regression analysis with *p* < 0.05 as the criterion to find possible AAGs with prognostic significances. The best combination for building a prognostic model was then further screened using a multivariate Cox regression. According to the median of the risk score, all patients were separated into high-risk and low-risk groups. To assess the difference in survival times between the high- and low-risk groups, Kaplan–Meier survival curves were created. ROC curves provided further assurance that prognostic significance was reliable.

### Construction and evaluation of nomogram

Age, stage, and risk score were shown to be independent determinants predicting the prognosis of HNSCC through univariate and multivariate Cox regression analysis. The “RMS” R program was used to combine these three variables, and the Cox method was used to create a nomogram. The predictive performance of the nomogram based on time-dependent ROC curves was evaluated using the “timeROC” R software. The possibility that the actual findings would match the projected outcomes was calculated using the concordance index (C-index). The most accurate prediction is shown by the 45-degree line. Through decision curve analysis (DCA), the nomogram’s clinical value was evaluated.

### Gene set enrichment analysis of the high-risk and low-risk prognostic groups

The samples were split into high-risk and low-risk groups in accordance with the aforementioned prognostic risk categorization. We conducted gene set enrichment analysis (GSEA) on the two sets of samples using GSEA 4.0.1 software to investigate the biological function and pathway enrichment of AAGs in various risk groups. GSEA made use of the Kyoto Encyclopedia of Genes and Genomes (KEGG) and Gene Ontology (GO) gene collections.

### Sensitivity analysis of immunotherapy and conventional therapy

To predict the effect of immunotherapy in patients with different risk groups, the immune response of patients was evaluated by calculating the TIDE score, IPS score, and MSI score. In addition, recognized techniques were employed to determine the immune cell infiltration level in HNSCC samples, including TIMER, CIBERSORT, XCELL, QUANTISEQ, MCPCOUNTER, EPIC, and CIBERSORT algorithms. Using the GDSC database, the IC50 values of 24 standard medicines were estimated to forecast their therapeutic effects on various risk subtypes.

### Mutation analysis of prognostically relevant AAG genes

The “RCircos” algorithm was used to pinpoint the locations of copy number variation (CNV) alterations in relevant AAGs on 23 chromosomes. To assess the differences in the mutational landscape between the high- and low-risk groups, somatic mutation data from the TCGA database was examined using the “maftools” software.

### Patient and tissue collection

Clinical Medical Research Ethics Committee of the First Affiliated Hospital of Anhui Medical University authorized the project (Quick-PJ 2023-05-30). The HNSCC tissues were given by the Department of Otolaryngology, Head and Neck Surgery of the First Affiliated Hospital of Anhui Medical University. According to WHO guidelines, all patients were diagnosed with HNSCC and underwent surgery. The surgical specimen is immediately immersed in liquid nitrogen for fast freezing before being stored in -80 degrees refrigerator. All participating patients gave their informed consents. All research was performed in accordance with relevant guidelines. Research involving human research participants have been performed in accordance with the Declaration of Helsinki.

### Immunohistochemistry (IHC) staining

Paraffin-embedded sections of tissue were deparaffinized in xylene and rehydrated through graded alcohols. Antigen retrieval was achieved by incubating the slides in 0.01M citrate buffer (pH 6.0) at 95℃ for 20 min. The slides were then cooled at room temperature for 20 min and rinsed with distilled water. After washing with phosphate buffered saline (PBS), the slides were incubated with 3% hydrogen peroxidase solution for 10 min to block endogenous peroxidase activity. The slides were then washed with PBS and incubated with primary antibody (1:200) overnight at 4℃. After washing with PBS, the slides were incubated with secondary antibody (1:1000) for 30 min at room temperature, followed by washing with PBS and incubation with streptavidin–horseradish peroxidase complex for another 30 min at room temperature. Finally, the slides were washed with PBS and visualized using diaminobenzidine tetrahydrochloride substrate solution for 5 min. Counterstaining was performed using hematoxylin. The slides were then dehydrated through graded alcohols and xylene and mounted with Permount. An Olympus CX41 fluorescent microscope was used to visualize staining. Image J software was used to evaluate the results. SERPIAN5, OLR1, PDGFA, S100A4, and APOH primary rabbit antibody were purchased from Abcam (Cambridge, USA), MSX1 primary rabbit antibody were purchased from Bioss (Beijing, China), secondary antibody (goat anti-rabbit immunoglobulin) were purchased from Abcam (Cambridge, USA).

### Western blotting

Protein was isolated from tissue using RIPA buffer. A NanoDrop 2000 (Thermo, USA) was used to measure the protein concentration. The protein samples were separated using SDS‒PAGE. After the protein was transferred to a PVDF membrane, a 3-h incubation in 5% skim milk at room temperature was performed to block nonspecific protein sites. Then, primary antibody was incubated with the membrane overnight. The PVDF membrane was then treated with the secondary antibody. Finally, an ECL luminescence kit (Thermo Scientific, USA) was used to visualize the protein on the PVDF membrane and capture images. Three separate experiments were performed, and the protein expression was analyzed using ImageJ each time. SERPIAN5, OLR1, PDGFA, S100A4 and APOH primary rabbit antibody were purchased from Abcam (Cambridge, USA), MSX1 primary rabbit antibody were purchased from Bioss (Beijing, China), secondary antibody (goat anti-rabbit immunoglobulin) were purchased from Abcam (Cambridge, USA).

### Reverse transcription quantitative PCR (RT‒qPCR)

To isolate RNA from tissue, TRIzol reagent was employed. The cDNA template was generated using the reverse transcription kit provided by QIAGEN (Japan). To evaluate the mRNA expression levels of SERPIAN5, OLR1, PDGFA, S100A4, APOH, and MSX1, a cDNA mixture was applied to a 96 well microplate and amplified according to standardized procedures. The 2 − ΔΔCt method was employed to determine the relative expression of mRNAs. The primer sequences utilized in the experiment are presented in Table 1.

**Table 1:** Sequences of primers used for RT-qPCR

Gene	Forward	Reverse
MSX1	5'-ACTCCTCAAGCTGCCAGAAGAT-3'	5’-TTACGGTTCGTCTTGTGTGTGTGC-3’
OLR1	5’-CTTTGGATGCCAAGTTGCTGAA-3’	5’-GCATCAAAGGAGAACCGTCC-3’
PDGFA	5’-TCCGCTAACTTCCTGATCT-3’	5’-CTTTCAACTTCGCCTTCTT-3’
S100A4	5'-CAGATCCTGACTGCTGCCATGGCG-3’	5’-ACGTGTCTGAAGGAGCCATGGTGG-3’
SERPINA5	5’-AGCAATGCGGTCGTGAT-3’	5’-TCCGGTCCAGGAGGTAG-3’
APOH	5ʹ-GCGCTCATCTTCTTTTCTGC-3ʹ	5ʹ-AGGGAACAACCACAGCAAAC-3ʹ
GAPDH	5′-GTGCTGAGTATGTCGTGGAGTCT -3′	5′-ACAGTCTTCTGAGTGGCAGTGA -3′

### Statistical analysis

To compare the variation between two variables, the t-test or Wilcoxon test was applied. We utilized the Kaplan–Meier technique and the log-rank test (two-stage test was employed when the curves were crossed) to evaluate differences in survival (Li et al. 2015). To handle, analyze, and display data, R software (version 4.1.2) and its related software packages were employed. The threshold for statistical significance was established as *p* < 0.05.

## Results

### HNSCC patients were separated into two groups based on clinical characteristics and survival rates

**Fig. 1:**
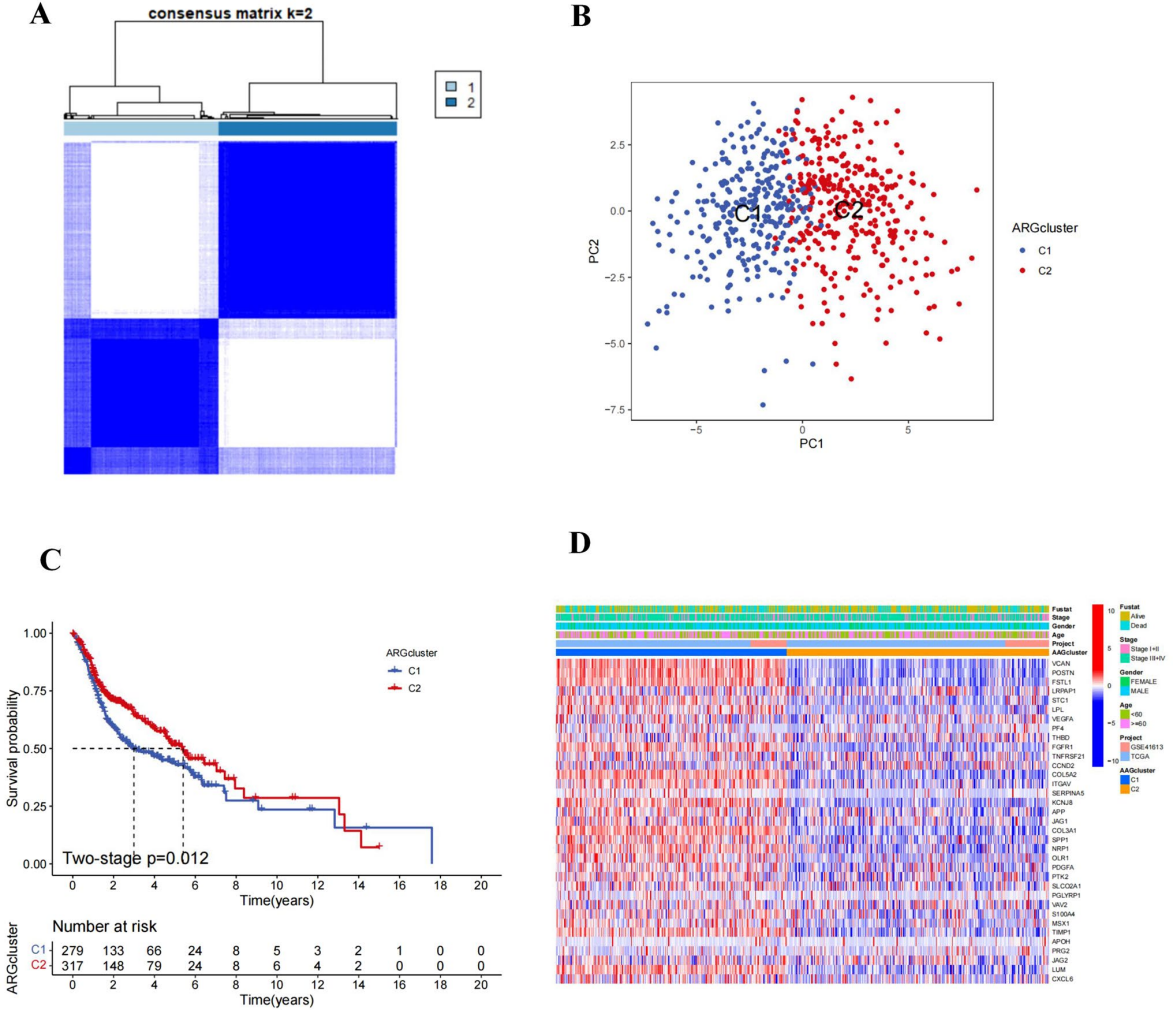
Consensus clustering analysis of HNSCC patients based on AAGs expression patterns. **A** Consensus matrix heatmap defining two clusters (*k* = 2) and their correlation area. **B** PCA analysis indicating an obvious difference in transcriptomes between the two subgroups. **C** Survival analysis of different subtypes. **D** Differences in clinicopathologic characteristics and expression levels of AAGs between the two distinct subgroups

The relationship between angiogenesis and tumorigenesis was investigated using 596 HNSCC patients from the TCGA-HNSCC and GSE41613. The combat function eliminates batch effects in the dataset. The batch effects before and after removal were shown in Supplementary Figs S1A and S1B, respectively. We used consensus clustering analysis to categorize HNSCC patients according to AAGs expression levels, to ascertain the association between AAGs expression patterns and HNSCC. The HNSCC patients in the overall cohort were evenly distributed in clusters C1 (*n* = 279) and C2 (*n* = 317), which showed that the ideal clustering variable was 2 (Fig. 1A). A favorable distribution across clusters was also supported by the PCA analysis’ findings (Fig. 1B). We observed the OS of the two clusters and found that there was a substantial difference in survival (Fig. 1C). In addition, as shown in Fig. 1D, we discovered statistical differences in the expression of AAGs and clinicopathological factors across the two clusters (Fig. 1D).

### Differences between clusters in biological function and tumor microenvironment

**Fig. 2:**
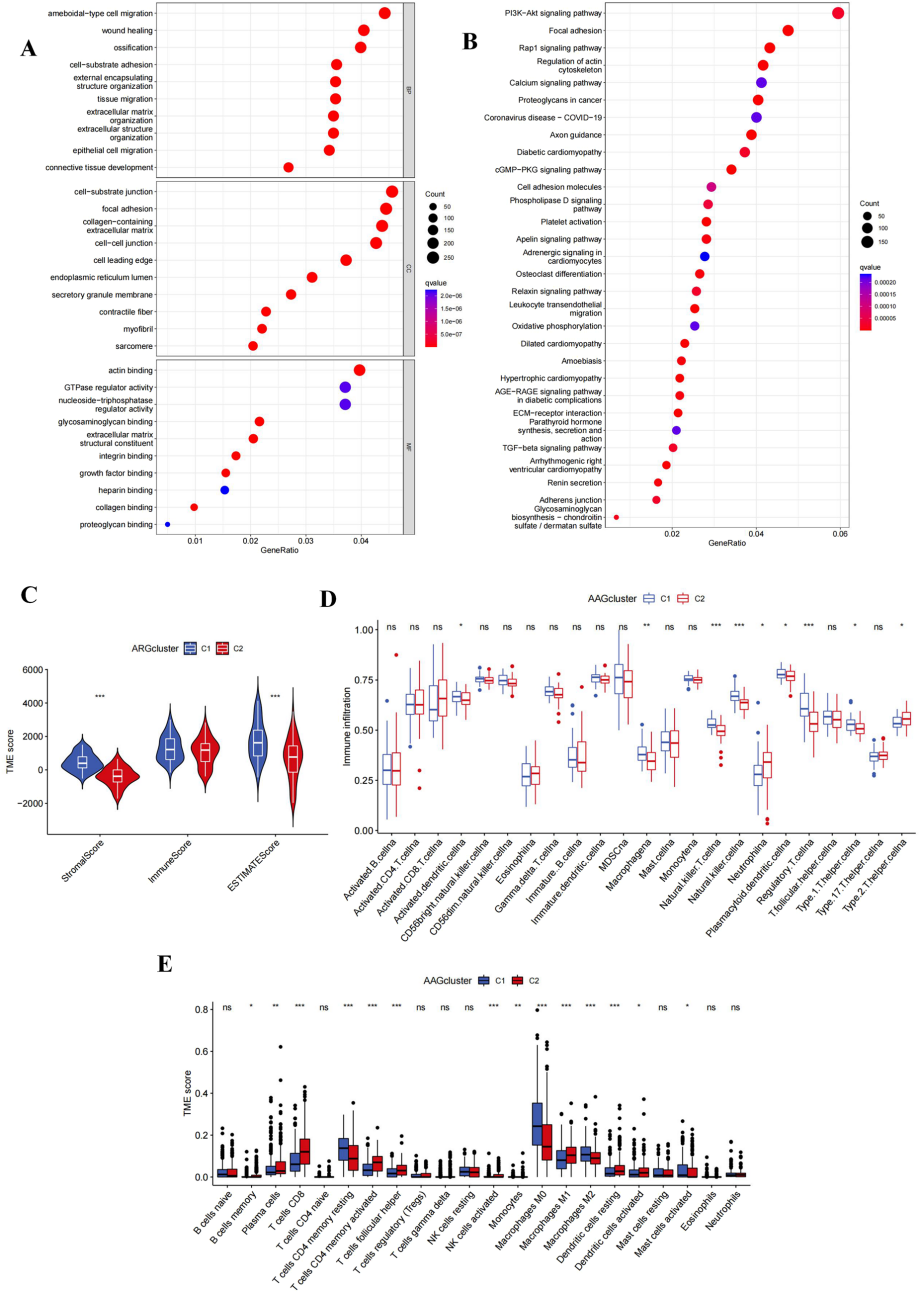
HNSCC clusters based on angiogenesis-related DEGs. **A**, **B** GO and KEGG enrichment analysis of DEGs in two HNSCC clusters; **C** Two HNSCC clusters Correlation between group and TME score; **D** ssGSEA calculated the abundance of 23 infiltrating immune cell types in two HNSCC clusters; **E** CIBERSORT calculated the correlation of 22 immune cells and TME scores between cluster 1 and cluster 2 patients Difference (*p < 0.05; **p < 0.01; ***p < 0.001)

Using the “limma” software, we acquired differentially expressed genes (DEGs) associated with angiogenic clusters and carried out functional enrichment analysis to observe the probable biological activities of angiogenic subclusters. Major biological processes involving DEGs included amoeba-type cell migration, wound healing, ossification, and cell-substrate adhesion. In terms of cellular components, DEGs were mainly enriched in cell-substrate junctions, focal adhesions, collagen-containing extracellular matrix, and cell–cell junctions. In terms of molecular function, DEGs were associated with actin binding, GTPase regulatory activity, and nucleoside-triphosphatase (Fig. 2A). KEGG analysis revealed abundant cancer and metastasis-related pathways, including PI3K-Akt signaling, focal adhesions, and Rap1 signaling, implying that angiogenesis is a key factor regulating tumor metastasis (Fig. 2B).

Immune and stromal element abundance in the TME could be evaluated by TME scores. To determine the association between AAGs and TME in HNSCC, we used the ESTIMATE algorithm to calculate TME scores in several clusters, including Stromal scores, Immune scores, and Estimated scores, The results demonstrated that cluster 1 patients had higher TME scores (Fig. 2C). In addition, we used ssGSEA methods to estimate the diversity of immune cell abundance. Activated dendritic cells, macrophages, plasmacytoid dendritic cells, NKT cells, NK cells, regulatory T cells, and type 1 T helper cells had significantly greater enrichment levels in cluster 1 than in cluster 2, but neutrophils and type 2 T helper cells had the reverse pattern (*p* < 0.05) (Fig. 2D). Meanwhile, CIBERSORT discovered that immune cells linked to increasing tumor growth and inflammation, including resting CD4 + T cells, resting M0macrophage, anti-inflammatory M2macrophage,and activatedmast cell was considerably concentrated in cluster 1 (Fig. 2E). These findings supported the finding that clustering based on AAGs expression identified a distinct subtype of HNSCC that was linked to immunological evasion, poor prognosis, tumor growth, and metastasis.

### Differences in immune checkpoints and HLA between clusters

**Fig. 3:**
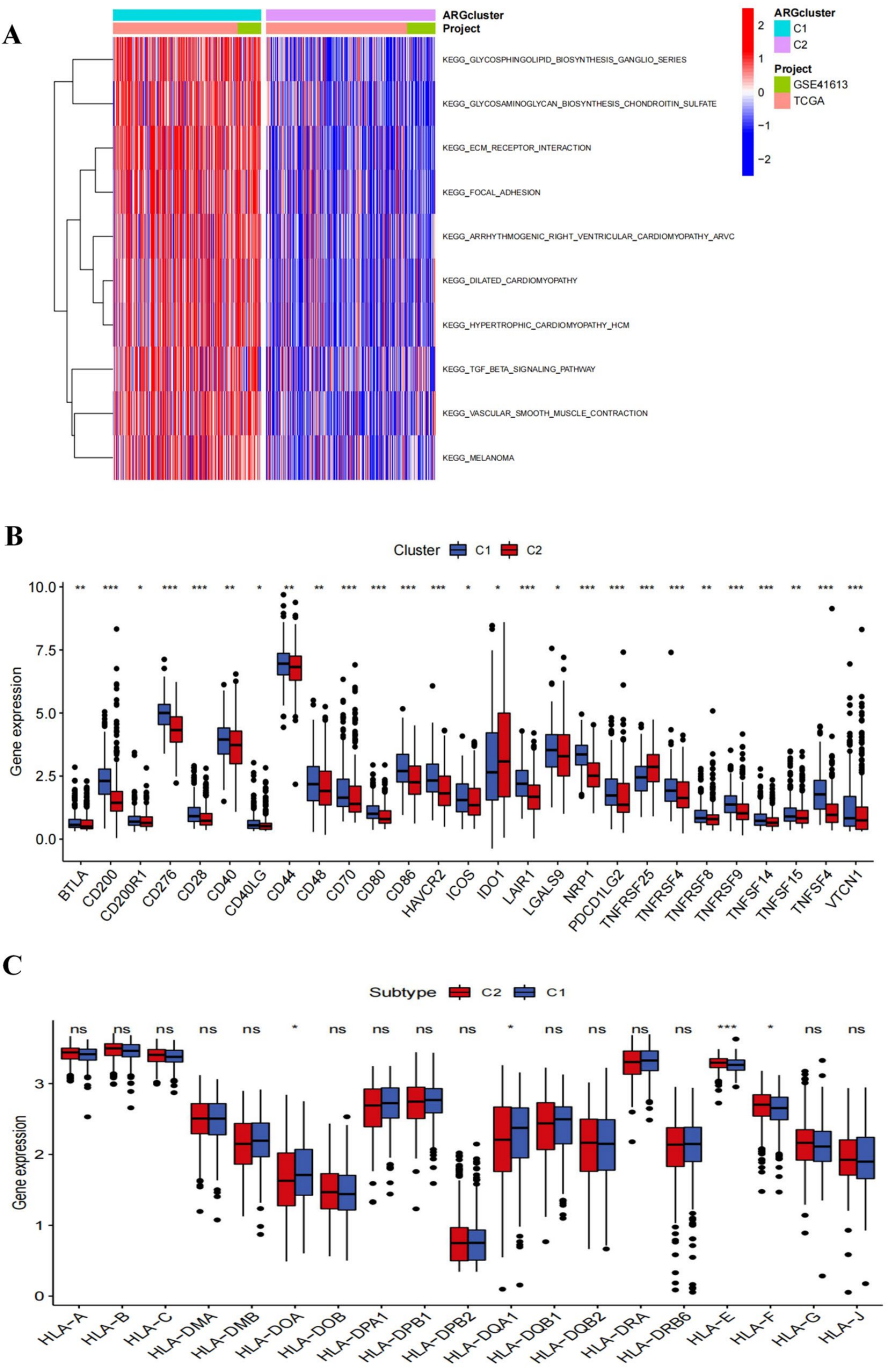
Analysis of pathway enrichment and TME differences between two different clusters. **A** GSVA analysis between two different clusters; **B** Expression difference analysis of immune checkpoints in cluster 1 and cluster 2; **C** Expression difference analysis of HLA molecules in two HNSCC clusters (*p < 0.05; **p < 0.01; ***p < 0.001)

The GSVA analysis revealed that cluster 1 had a high proportion of metastasis-related pathways (glycosphingolipid biosynthesis ganglia severe, ECM receptor interaction, and focal adhesions) as well as tumor-related pathways (TGF-β signaling system, melanin) (Fig. 3A). The expression of immunological checkpoints and HLA varied between clusters was also evaluated. The findings demonstrated that cluster 1 has highly expressed immunosuppressive sites such as BTLA, CD200, CD200R1, CD44, HAVCR2, etc. that promote immunosuppression and tumor formation (Fig. 3B). HLA-E and HLA-F, non-canonical HLA-I antigens, were considerably overexpressed in cluster 1, while HLA-DOA and HLA-DOA1 expression levels in cluster 1 were lower than in cluster 2 (Fig. 3C).

### An HNSCC diagnostic model based on AAGs was constructed and validated

**Fig. 4:**
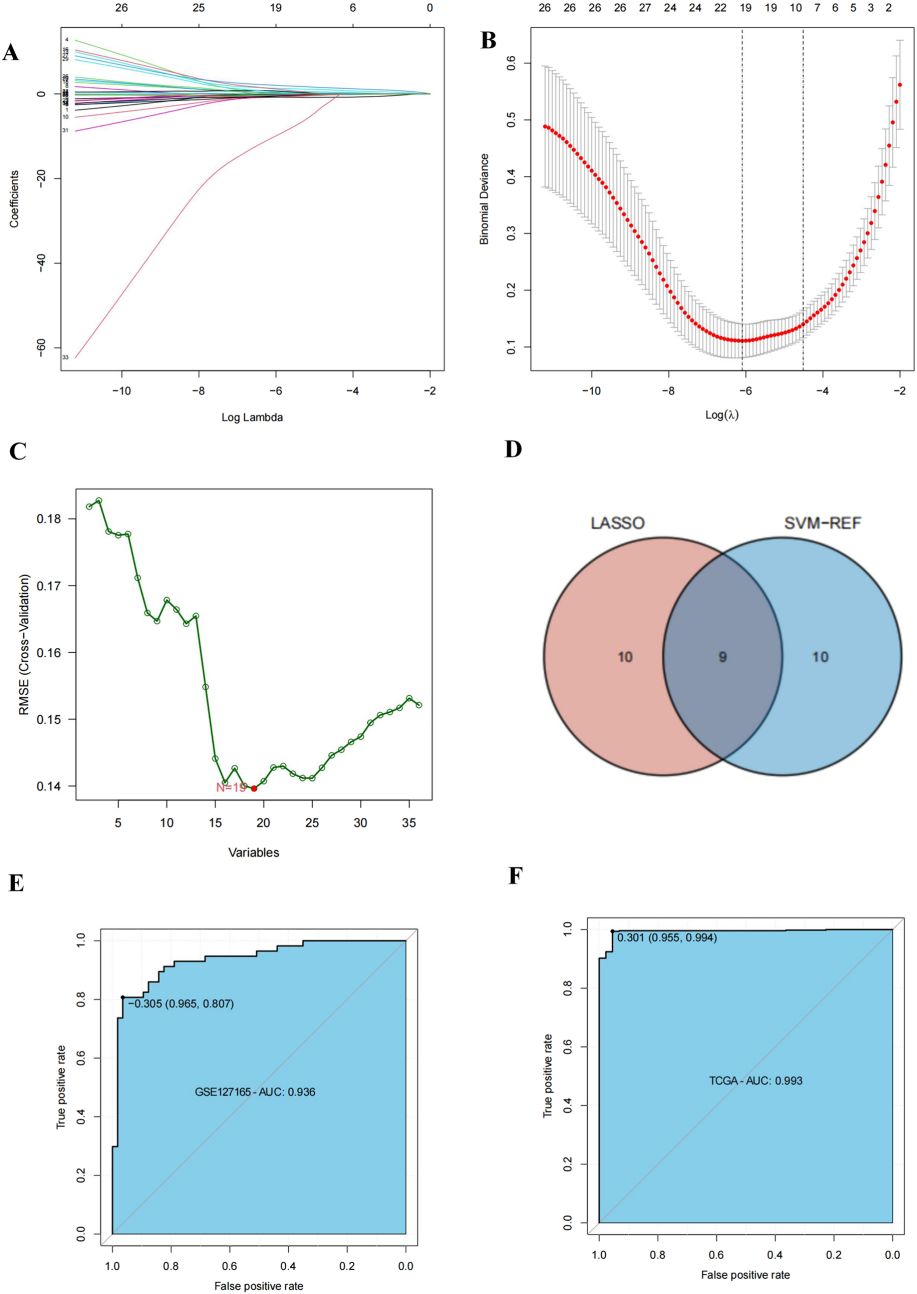
Construction and validation of HNSCC diagnostic signature. **A**, **B** Multivariate least absolute shrinkage **A** and selection operator (LASSO) **B** Regression analysis; **C** Support vector machine analysis; **D** The intersection of the genes screened by SVM and multivariate lasso analysis was used to construct the diagnostic signature; **E** Sensitivity and specificity of the ROC curve based on the TCGA dataset; **F** Sensitivity and specificity of the ROC curve derived based on the GSE14520 dataset

We created a diagnostic model to investigate the relevance of AAGs in the diagnosis of HNSCC. For model accuracy and refinement, we performed LASSO (Fig. 4A, B) and SVM (Fig. 4C) analyses, and selected 9 genes from intersections (Fig. 4D) to construct a diagnosis model using multiple logistic regression analysis. The equation is = (0.8542 × VEGFA) + (1.2981 × COL5A2) + ( – 2.2259 × SERPINA5) + (0.4481 × KCNJ8) + (0.2258 × JAG1) + (0.4377 × SPP1) + (-0.2502 × PTK2) + (2.6047 × VAV2) + (-0.2570 × MSX1). The receiver operating characteristic curve (ROC) for the TCGA dataset displayed excellent area under curve (AUC) values (0.993), specificity (0.955), and sensitivity (0.994) (Fig. 4E). In the independent dataset GSE127165, we additionally verified the diagnostic model mentioned above (Fig. 4F).

### An HNSCC prognostic signature based on AAGs was constructed and validated

**Fig. 5:**
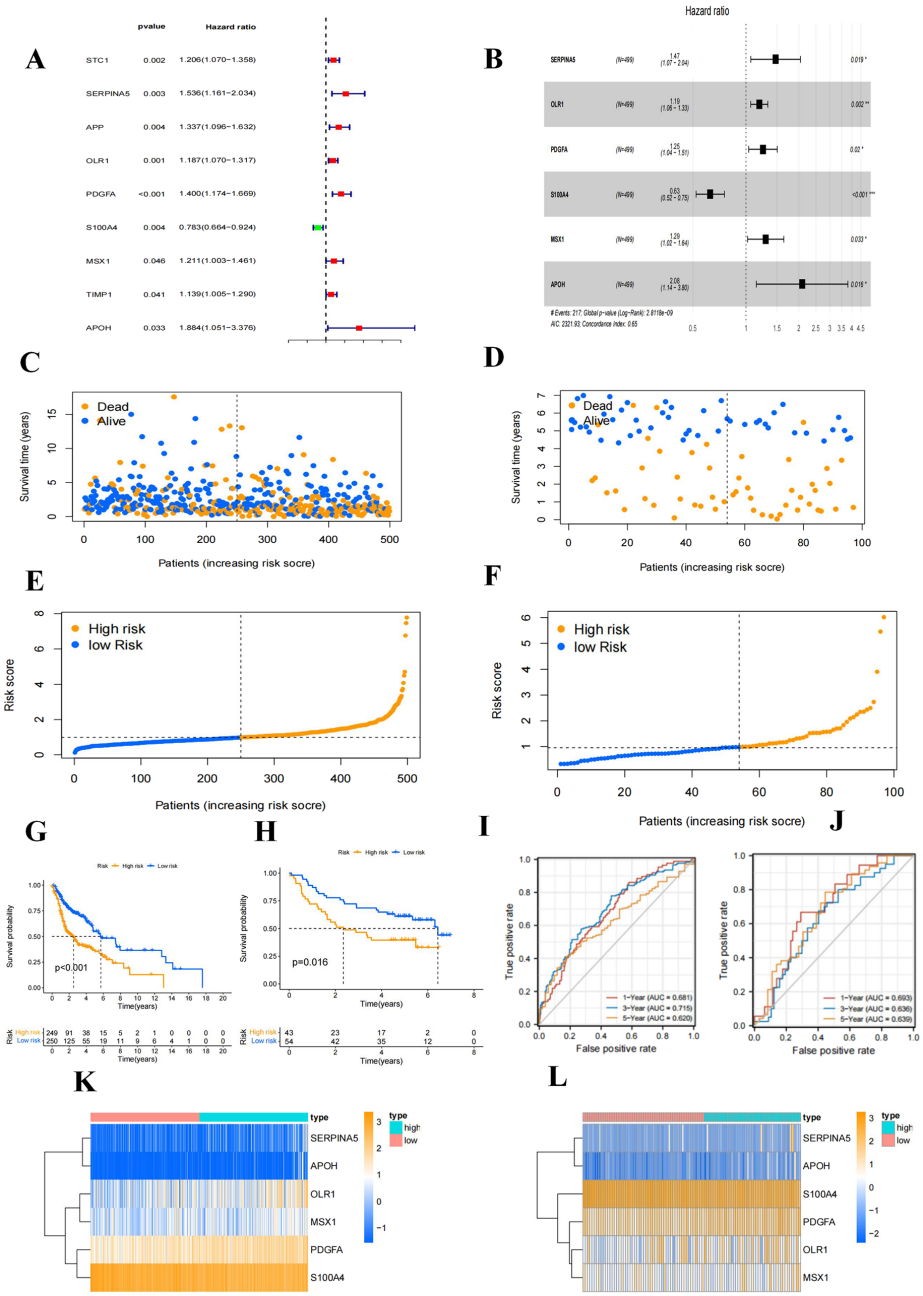
Construction of HNSCC prognostic signature. **A** Forest plot showed that the prognosis related AAGs were preliminarily obtained by univariate regression analysis; **B** Forest plot showed that the AAGs used to construct the prognosis model was further determined by multivariable regression analysis; **C**, **D** Scatter plot of risk score distribution and patient survival status in the training and validation cohorts, respectively; **E**, **F** Ranking plot and median value of risk score in the training and validation cohorts, respectively; **G**, **H** Kaplan–Meier analysis comparing OS between high- and low-risk groups in the training and validation cohorts, respectively; **I**, **J** ROC curves reflecting the sensitivity and specificity of the signature for predicting 1-year, 3-year and 5-year survival in the training and validation cohorts, respectively; **K**, **L** Expression pattern comparison of the six selected prognostic genes used to construct the signature between the high- and low-risk groups in the training and validation cohorts, respectively

Nine differentially expressed AAGs (STC2, SERPINA5, APP, OLR1, PDGFA, S100A4, MSX1, TIMP1, APOH) were shown to be significantly linked with OS in HNSCC patients by univariate cox regression analysis (Fig. 5A). The impact of nine AAGs associated with prognosis on OS in HNSCC patients was reevaluated using multivariate cox regression analysis. The HNSCC prognostic model was created using six AAGs with independent predictive significance (SERPIAN5, OLR1, PDGFA, S100A4, MSX1, and APOH) (Fig. 5B). The training cohort (TCGA dataset, *n* = 499) and validation cohort (GSE41613 dataset, *n* = 97) were used to validate the prognostic value of the generated signatures. Overall, both in the training cohort (Fig. 5C) and validation cohort (Fig. 5D), the number of fatalities rose with higher risk grades. Using the median risk score, samples were split into high- and low-risk groups **(**Fig. 5E, F). According to Kaplan–Meier survival analysis, patients in the high-risk group had substantially poorer OS than those in the low-risk group both in the training and validation cohorts (*p* < 0.001) (Fig. 5G, H). The AUC values of the 1-year, 3-year, and 5-year ROC curves of the constructed signatures in the training cohort were 0.681, 0.715, and 0.620, respectively (Fig. 5I); the AUC values of the 1-year, 3-year, and 5-year ROC curves of the constructed signatures in the validation cohort were 0.693, 0.636, and 0.639, respectively (Fig. 5J). These results demonstrated the accuracy of the signature constructed based on the six AAGs for HNSCC prognosis prediction. Expression patterns of the six prognostic genes that were chosen and utilized to build the signature between the high- and low-risk groups in the training and validation cohorts were shown in Fig. 5K and L.

### AAGs score clinical correlation analysis

We explored the association between AAG risk score and different clinicopathological variables (age, sex, grade, stage, N stage, T stage, and HPV infection) (Fig. S1C) and found that greater stages and T stages were statistically linked to higher risk scores. Using univariate (Fig. S1D) and multivariate Cox regression analysis (Fig. S1E), we also investigated the independence of various clinicopathological variables for prognosis prediction. The findings indicated that in the TCGA-HNSCC cohort, age, stage, and risk score were independent prognostic variables. In addition, patients were separated into different subgroups according to clinical criteria to further investigate the impact of risk score on the prognosis of HNSCC patients. In all clinical parameter groupings, high-risk patients had poorer survival rates than low-risk patients (Fig. S2).

### Constructing a nomogram to predict patient outcomes

**Fig. 6:**
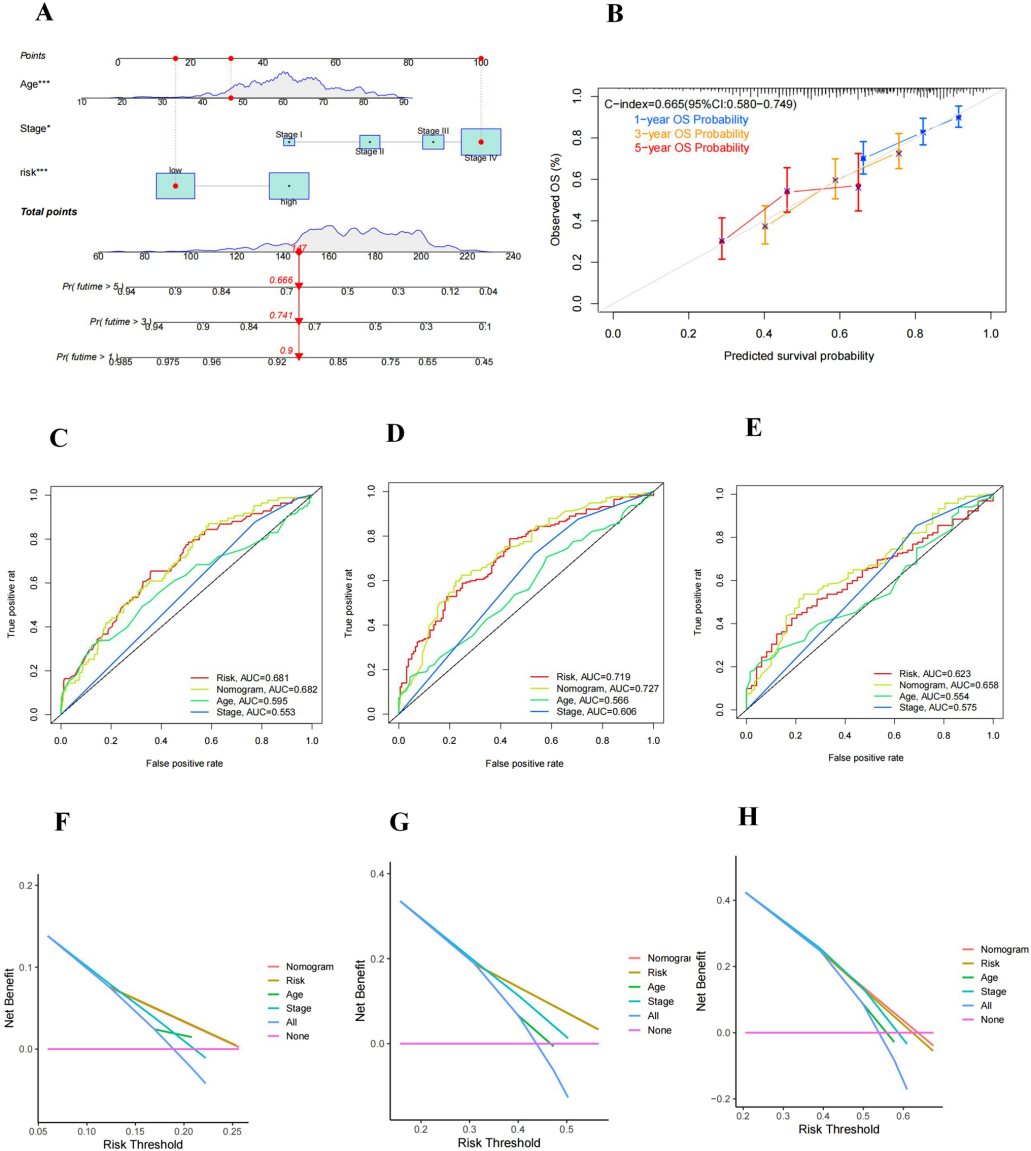
Construction and validation of a nomogram. **A** Construction of the nomogram. **B** 1-, 3-, and 5-year ROC curves of the nomogram in the entire cohort. **C**–**E** ROC curves of the nomogram and each independent prognostic factor for 1-, 3-, and 5-year OS, respectively. **F**–**H** DCA curves of the nomogram and each independent prognostic factor for 1-, 3-, and 5-year OS, respectively

We further constructed a prognostic nomogram for HNSCC by combining age, stage, and risk score (Fig. 6A). The calibration curves were produced, and the results demonstrated a high agreement between the actual observed and anticipated values when the nomogram was used to measure 1-, 3-, and 5-year OS in HNSCC patients (Fig. 6B). The AUC values of the nomogram were considerably greater than those of each independent prognostic factor (Fig. 6C–E). In addition, we also discovered that this prognostic nomogram constructed based on different clinical parameters had greater net gains in forecasting prognosis (Fig. 6F–H).

### Biological processes and pathways in various risk groups

**Fig. 7:**
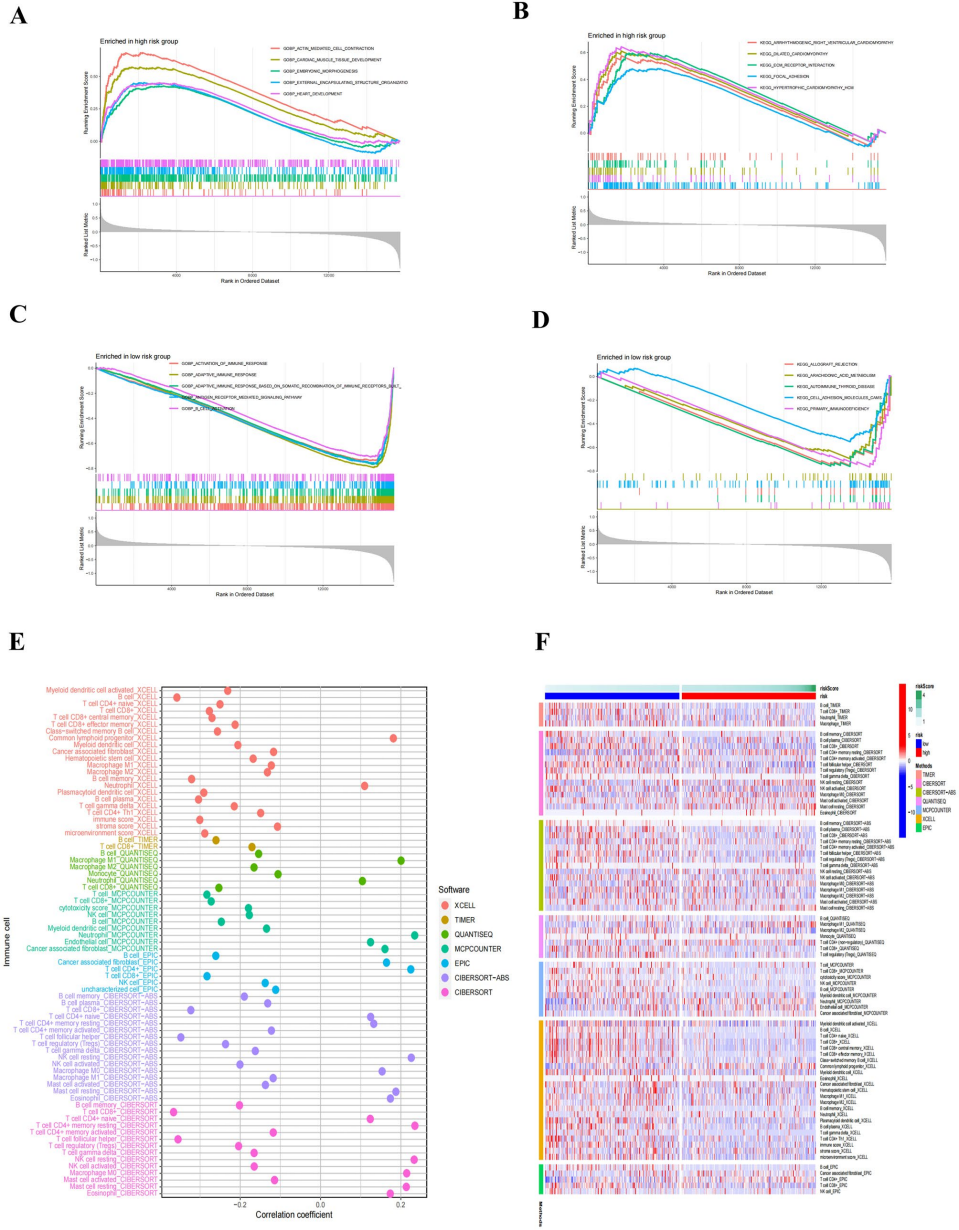
Gene set enrichment analysis identifies biological pathways and processes associated with risk scores within the TCGA cohort, while multiple algorithms determined levels of multiple immune cell infiltration associated with risk scores. **A** GSEA analysis of the high-risk group based on the GO database; **B** GSEA analysis of the high-risk group based on the KEGG database; **C** GSEA analysis of low-risk groups based on GO database; **D** GSEA analysis of low-risk group based on KEGG database; **E**, **F** The relationship between AAG risk score and immune cell infiltration in HNSCC samples, using Timer, CiberSort, Xcell, QuantieQ, MCPCounter, EPIC, and CiberSor algorithms

GSEA was used to compare high-risk and low-risk group in TCGA cohort to understand the influence of risk score on HNSCC associated biological processes. The results showed that high-risk group predominantly enriched the pathways linked to cardiac development, myocardial tissue development and myocardial dysfunction (Fig. 7A, B). The low-risk group predominantly enriched immune system activation and immune response pathways, such as GO (Activation of immune response, Adaptive immune response, Adaptive immune response based on immune somatic recombination, Antigen receptor mediated signal transduction pathway, B cell activation) (Fig. 7C) and KEGG (Allograft rejection, Autoimmune thyroid tissue, Immune deficiency) (Fig. 7D).

### Immune cell infiltration and immunotherapy response in different risk groups

To further investigate the tumor immune responses of HNSCC patients based on risk scores, we evaluated the infiltrating immune cells in the TME of HNSCC patients using seven algorithms. The AAG score was inversely linked with the infiltration of several anti-tumor immune components. The majority of immune cells, including CD8 + T cells, activated dendritic cells, and natural killer (NK) cells, were increased in the low-risk group relative to the high-risk group (Fig. 7E, F).

**Fig. 8:**
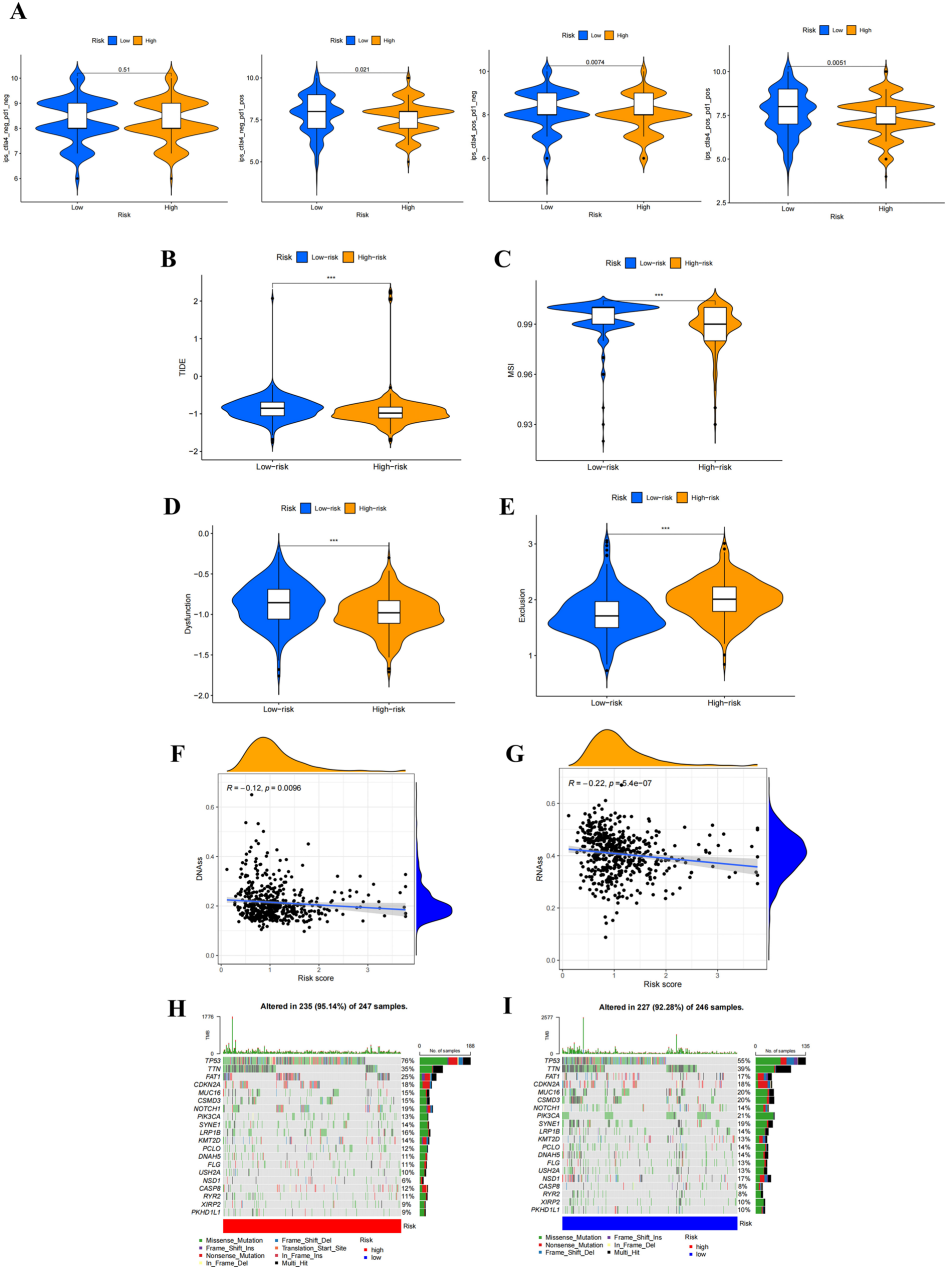
Association between AAG-based risk scores and immunotherapy sensitivity. **A** IPS, IPS-PD-1 blocker, IPS-CTLA-4 blocker, IPS-CTLA-4 and PD-1 blocker scores between high- and low-risk groups; **B** TIDE scores between high- and low-risk groups, respectively; **C** MSI scores between high- and low-risk groups; **D** T cell dysfunction scores between high- and low-risk groups; **E** T cell exclusion scores between high- and low-risk groups; **F** Correlation between AAG score and mDNAsi stemness index; **G** Correlation between AAG score and mRNAsi stemness index; **H**, **I** The mutational profiles of HNSCC patients between different risk groups. (*** *p* < 0.001)

Multiple studies have demonstrated that an immunogenicity score (IPS) is an excellent predictor of ICI response (Liu et al. 2023). The main immunological checkpoints are CTLA-4, PD-1, PD-L1, and programmed death ligand 2 (PD-L2). Consequently, the scores of IPS, IPS-CTLA-4 blocker, IPS-PD-1 blocker, and IPS-CTLA-4 + PD-1 blocker were utilized to assess the prospective effect of ICIs. The results showed that there was no significant difference in IPS scores between the high- and low-risk groups. However, IPS-CTLA-4, IPS-PD-1, and IPS-CTLA-4 + PD-1 scores were considerably higher in the low-risk group, suggesting that the low-risk group is more immunogenic to ICIs (Fig. 8A).

Using TIDE, we subsequently analyzed the potential clinical effectiveness of immunotherapy in different risk subgroups. Higher TIDE scores indicating a greater possibility of immune evasion, indicating that patients are less likely to benefit from ICI treatment. The TIDE score of the high-risk subgroup was lower than that of the low-risk subgroup, indicating that high-risk patients were more likely to benefit from ICI treatment than low-risk patients (Fig. 8B). We also discovered that the low-risk subgroup had higher microsatellite instability (MSI) scores (Fig. 8C), greater T cell dysfunction scores (Fig. 8D), and lower T cell exclusion scores (Fig. 8E). Furthermore, we also evaluated the potential correlation of risk score and CSC score in HNSCC patients. The results showed that the lower the risk score in HNSCC patients, the higher the CSC score, the more prominent stem cell characteristics and the lower the cell differentiation level (Fig. 8F, G).

In addition, we also investigated differences in the distribution of somatic mutations in the AAG scoring patterns of HNSCC patients. As shown in the Fig. 8H, I, the mutation rates of TP53 and TTN in the high-risk group and low-risk group were both over 20%, the mutation rates of FAT1 in the high-risk group were over 20%, and the mutation rates of MUC16, CSMD3, and PIK3CA in the low-risk group were greater than or equal to 20%.

### Conventional therapy response in different risk groups

**Fig. 9:**
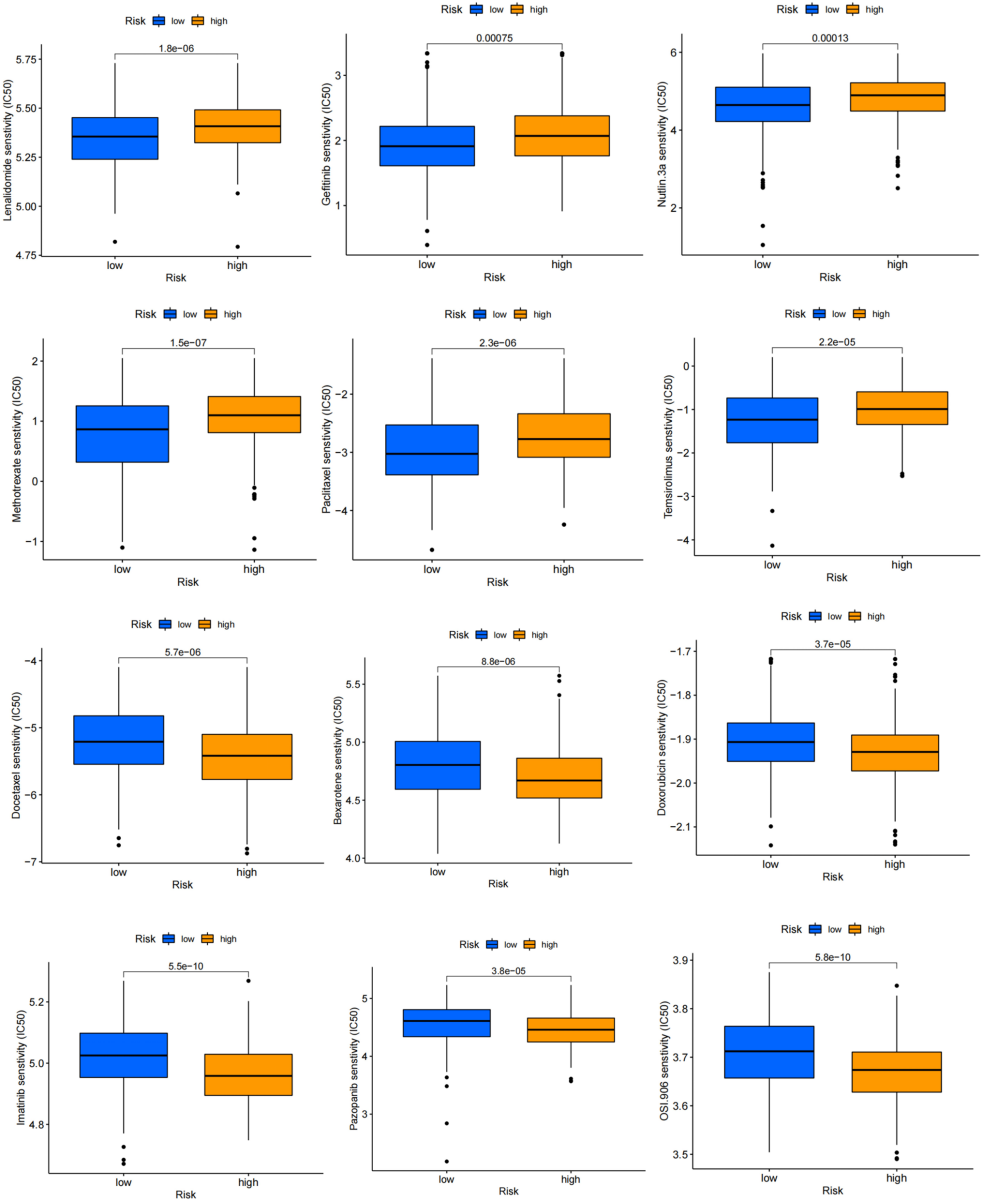
Comparison of IC50 of multiple anti-tumor drugs in high-risk and low-risk groups

We calculateed IC50 values for 24 medications in TCGA-HNSCC patients to evaluate the utility of the risk score as a biomarker for predicting response to conventional treatment in HNSCC patients. We found lower IC50 for Lenalidomide, Gefitinib, Nutlin.3a, Methotrexate, Paclitaxel, Temsirolimus etc. in low-risk patients, implying better response; whereas high-risk patients responded better to Docetaxel, Bexarotene, Doxorubicin, Imatinib, Pazopanib, OSI.906 etc. (Fig. 9). Taken together, these results showed an association between AAGs and drug sensitivity.

### Validation of the expression of six AGG genes in tissues

**Fig. 10:**
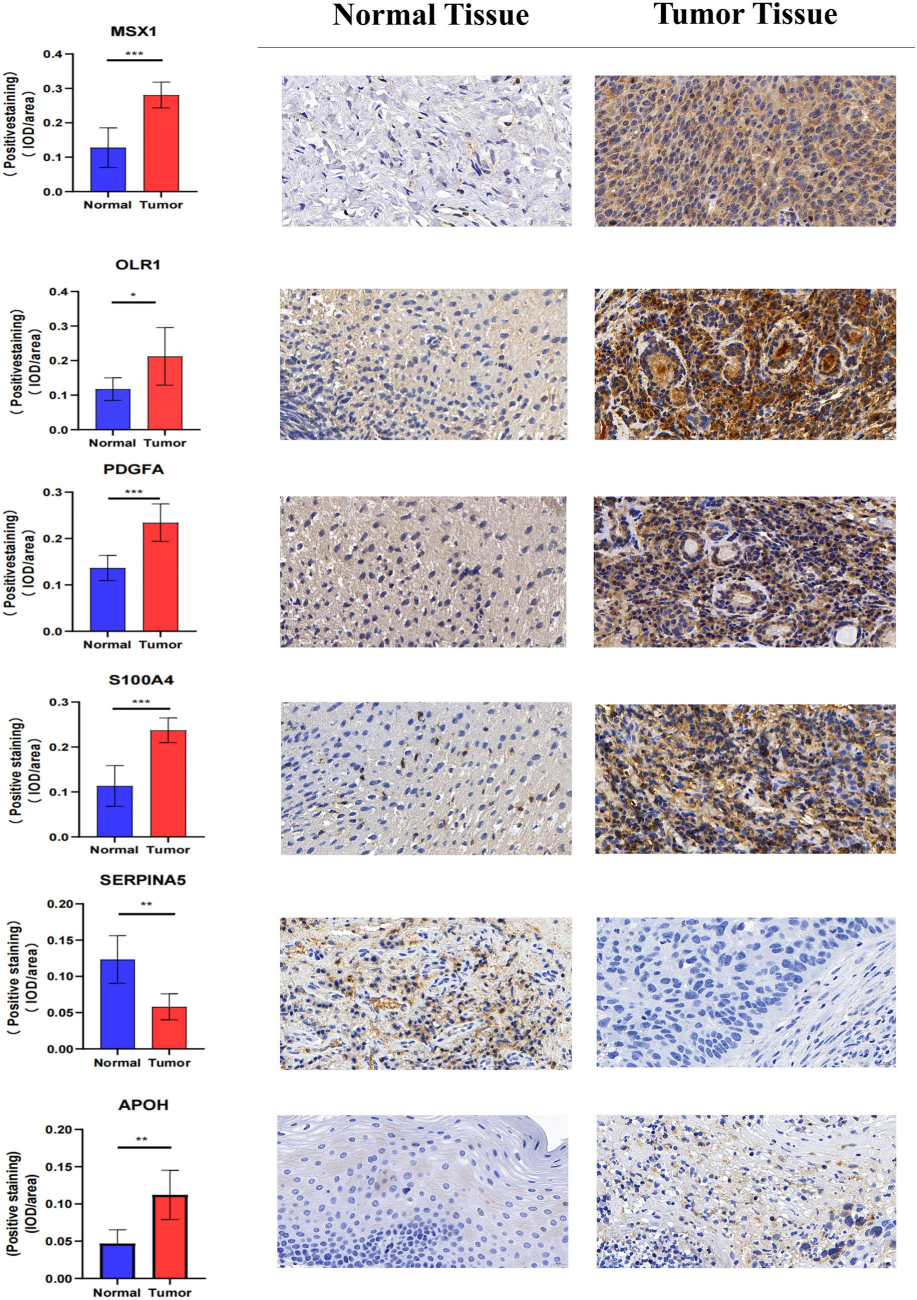
The IHC staining results showed that the expression levels of OLR1, PDGFA, S100A4, MSX1, and APOH in HNSCC tissues were higher than those in adjacent normal tissues, except for SERPIAN5, which was lower in HNSCC tissues than in adjacent normal tissues

**Fig. 11:**
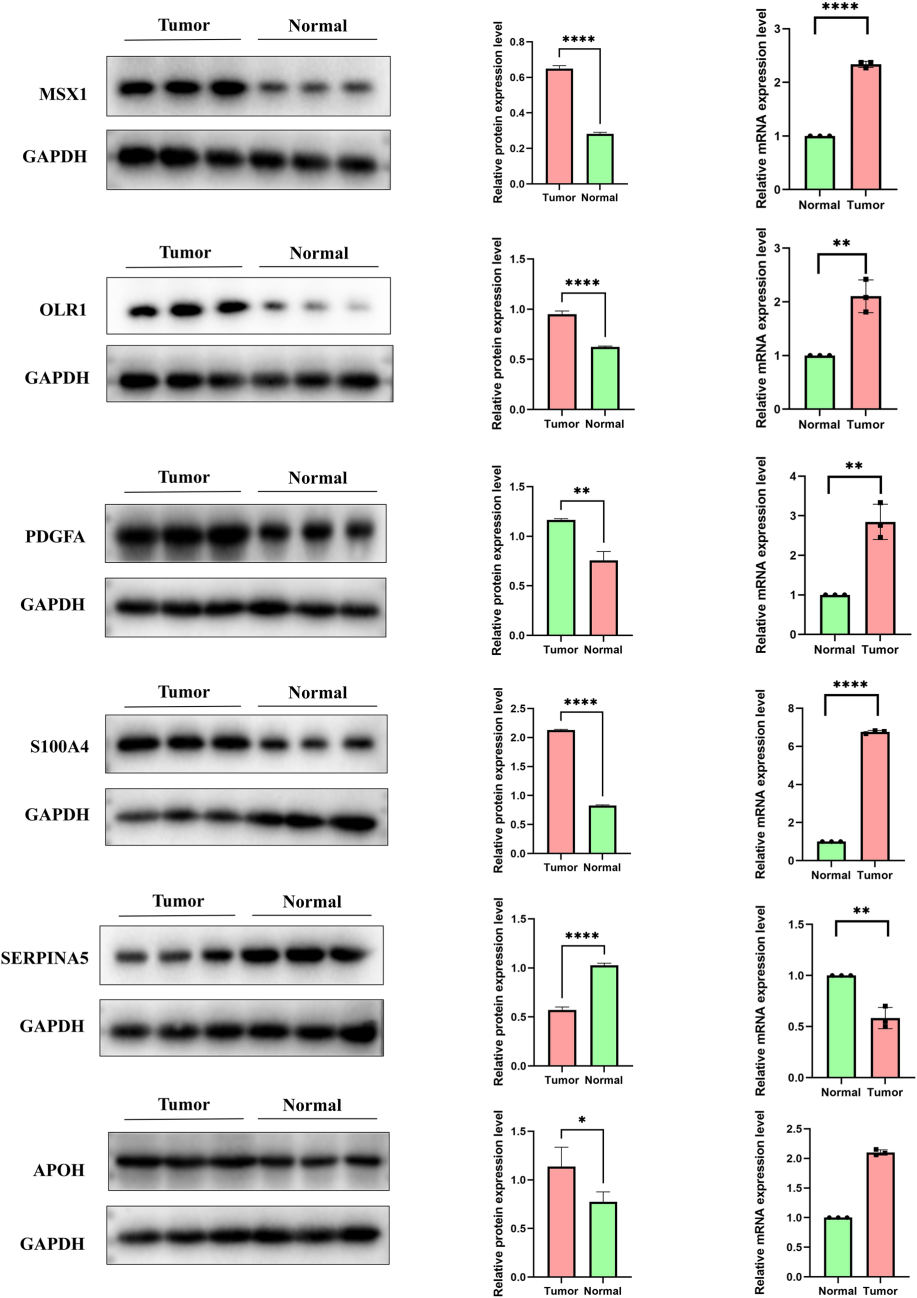
The western blotting and RT-qPCR results showed that the expression levels of OLR1, PDGFA, S100A4, MSX1, and APOH in HNSCC tissues were higher than those in adjacent normal tissues, except for SERPIAN5, which was lower in HNSCC tissues than in adjacent normal tissues

We collected six pairs of HNSCC tissues and their adjacent normal tissues, and detected the expression differences of the six AGG genes used to construct the prognostic model in HNSCC tissues and normal tissues. The IHC staining (Fig. 10), western blotting and RT-qPCR (Fig. 11) results found that except SERPIAN5, which was relatively low-expressed in HNSCC tissues, the other five genes (OLR1, PDGFA, S100A4, MSX1, and APOH) were all highly expressed in HNSCC tissues. Supplementary file 1 contains IHC staining results for all six pairs of tissues. Supplementary file 2 contains original whole membrane of western blotting bands.

## Discussion

Numerous studies have demonstrated that angiogenesis is an important factor in the occurrence, development, and migration of HNSCC (Vassilakopoulou et al. 2015; Ludwig et al. 2018). Angiogenic cytokines can activate or inhibit tumor progression through a variety of complex mechanisms (Farlow et al. 2021; Qing et al. 2022; Dufies et al. 2019). Immunosuppressive cells, such as Treg and tumor-associated macrophages, are activated by angiogenic cytokines, which also suppress immune effector cells and antigen-presenting cells. In turn, these suppressive immune cells may promote angiogenesis, which contribute to a vicious cycle with poor immune activation (Rahma and Hodi 2019). Targeting angiogenesis while immunotherapy has been shown to be an effective and feasible strategy to further expand the applicability of cancer therapy (Lee et al. 2020). However, many reports have only highlighted a single AAG or specific immune cell subtype. Therefore, it is necessary to further elucidate the overall impact and tumor immune characteristics under the action of different AAG combinations.

In this study, we used a consensus cluster analysis to identify two novel clusters (named cluster 1 and cluster 2) in HNSCC patients based on the expression differential of 35 AAGs. The dependability of the two clusters was further confirmed by PCA analysis. In the subsequent analysis of differences in survival and clinical characteristics, AAG expressions were found to be up-regulated and the survival rate was lower in cluster 1. These results showed that an increase in AAG expressions may be linked to poor survival and prognosis in HNSCC, and that anti-angiogenic therapy may have a better effect on cluster 1.

We also carried out GO/KEGG enrichment analysis of DEGs across clusters to further investigate the probable biological activities of angiogenic clusters. According to the findings of the GO enrichment analysis, angiogenesis-related DEGs were enriched in many important pathways that promote the migration of tumor cells, including amoebic cell migration, wound healing, ossification, cell–matrix junctions, and focal adhesions. According to the results of the KEGG enrichment analysis, DEGs were also significantly enriched in the functional pathways lead to tumor metastasis. Focal adhesions, ECM receptor interactions, and TGF-β signaling pathways identified in subsequent GSVA analysis further validated the above results. Interestingly, GSVA analysis also revealed that cardiac dysfunction related pathways were enriched in cluster 1.These results suggested that a specific cluster of HNSCC associated with myocardial disease was obtained by clustering, which showed higher tumor metastatic ability and spread. Thus, the identified AAG-related pathways add to the tumor biology information that further characterizes HNSCC.

Previous studies have shown that although some HNSCC patients achieve durable benefit from immunotherapy, while many HNSCC patients do not respond to immunotherapy from the beginning, and the exact mechanism has not been elucidated. Complex factors in the TME may be involved in this process. Therefore, we evaluated the TME between different subtypes. The findings revealed that cluster 1 had a much higher Immune score and Stromal score than cluster 2, and immune cells associated with tumor progression and inflammation also had a more significant infiltration level in cluster 1.

In addition, the immune checkpoints associated with tumor immunosuppression and immune escape were considerably more expressed in cluster 1 than in cluster 2. Among them, the high expression of CD200 is related to the immune escape and poor prognosis of malignant tumors (Jung et al. 2015; Shin et al. 2019), and the regulatory role of CD44 in cancer stem cells of HNSCC patients has been clarified in recent researches (Liu et al. 2023; Sharaf et al. 2021).

In the past few years, the mechanism of tumor cell resistance to ICIs has been extensively studied and malfunction of the antigen presentation mechanism through HLA-I antigens is a possible mechanisms (Kok 2020). In this study, we found that cluster 2 had much greater levels of HLA-E and HLA-F expression over cluster 1, and that the high expressions of non-classical HLA-I antigens in tumor tissues could not only mediate resistance to cytotoxic T lymphocytes (CTL), but also lead to tolerance to NK cell-mediated cytotoxicity (Ferris 2015; Gornalusse et al. 2017). The above results suggested that HNSCC clusters generated based on AAGs expression pattern have significant implications for guiding immunotherapy.

The diagnostic and prognostic role of AAG in HNSCC was next examined. SVM and LASSO analysis were used to identify relevant factors from the TCGA dataset, and logistic regression was used to create an HNSCC diagnosis model with nine AAGs. The specificity and sensitivity were 0.955 and 0.994 respectively. On the independent dataset GSE127165, we also successfully validated the diagnostic model with a specificity of 0.965 and a sensitivity of 0.807. These results showed that the diagnostic model has high reliability and stability. Small differences in model performance between the training and validation cohorts may be attributed to different sources of cohort inclusion.

In addition, we screened 6 AAGs (SERPIAN5, OLR1, PDGFA, S100A4, MSX1, APOH) which were significantly associated with HNSCC prognosis and established an prognostic risk signature. The AUC values of the 1-year, 3-year, and 5-year ROC curves in the validation set were 0.693, 0.636, and 0.639, respectively, while the corresponding values in the training cohort were 0.681, 0.715, and 0.620. These findings back up the accuracy of prognostic signature. To distinguish clearly between high-risk and low-risk groups, GSEA was employed. Our findings indicated that the low-risk group was connected with functional pathways related to immune system activation and immunological response, while the high-risk group was connected with functional pathways related to cardiac tissue development and cardiomyopathy. Functional differences between prognostic risk groups help to select relatively effective treatment options for different types of patients.

Moreover, we evaluated the effectiveness of immunotherapy in various prognostic risk categories using TIDE and IPS scores. T cytotoxic cell exclusion was found to be lower, while cytotoxic T cell dysfunction was higher in the low-risk group. Although there were more tumor-infiltrating CTL, their dysfunction was also more pronounced, potentially weakening the ability of cytotoxic T cells to kill cancer cells. Patients in low-risk group responded more positively to anti-PD-1 and anti-CTLA-4 treatment.

We found that a prominent feature of HNSCC is the high burden of tumor mutations. MSI results can reflect somatic mutations. Excessive somatic mutation suggests that the tumor produces more neoantigens, allowing it to elude immune surveillance. MSI score helps to predict the efficacy of immunotherapy to a certain extent, and the value of MSI in predicting the efficacy of PD-1 immunotherapy in colorectal cancer has been reported (Toh et al. 2016; Motta et al. 2021). Therefore, we used the MSI score to predict how immunotherapy would affect patients in different risk groups and found that low-risk patients had a considerably higher MSI score than high-risk patients, indicating that low-risk patients would gain more from immunotherapy.

Finally, by evaluating the sensitivity of multiple conventional drugs in different risk groups, 24 conventional drugs with significant differences in sensitivity were obtained, which will help to select appropriate drugs for different HNSCC subtypes and improve clinical efficacy.

Obviously, our research also has limitations, mainly reflected in the lack of real clinical cohorts to validate the results of bioinformatics analysis. Therefore, we are conducting a real-world clinical research and hope that the predictive model we constructed can truly be applied in clinical practice. In addition, we also conducted functional and mechanistic studies on relevant AAGs to explore their specific roles in the occurrence and development of HNSCC.

## Conclusion

In this study, we first analyzed the expression pattern of AGGs in HNSCC. Then, nine AAGs were screened, and a diagnostic model of HNSCC was constructed based on them. Next, six AAGs with independent prognostic value were obtained through further screening, and a prognostic risk signature of HNSCC was constructed. The validation cohort confirmed that this prognostic risk signature performed well in the prognosis prediction of HNSCC. It is worth noting that it also has guiding significance in tumor immunity, signaling pathways, drug response, etc. Moreover, we combined the prognostic risk score, age and stage to further construct the HNSCC prognostic nomogram. In conclusion, our study provides practical clinical help for the diagnosis, treatment and prognosis prediction of HNSCC.

### Supplementary Information

Below is the link to the electronic supplementary material.

Supplemental Figure S1 (A-B) The batch effects before and after elimination, respectively; (C) The relationship between AAG score and different clinicopathological features (age, sex, grade, stage, N stage, T stage, and HPV infection),; (D-E) Univariate and multivariate Cox regression analysis sh owed that age, stage and constructed risk score were independent prognostic factors in the TCG A HNSCC cohort. Supplementary file1 (JPG 2953 KB)

Supplemental Figure S2 High-risk patients had lower survival rates than low-risk patients in all subgroups of clinical parameters (age, sex, grade, stage, N stage, T stage and HPV infection). Supplementary file2 (JPG 3565 KB)

Supplementary file 1 IHC staining results for all six pairs of tissues. Supplementary file3 (PDF 22085 KB)

Supplementary file 2 Original whole membrane of western blotting bands. Supplementary file4 (PDF 596 KB)

## Data Availability

The datasets generated during and/or analysed during the current study are available from the corresponding author on reasonable request.
