# Supplementary material for: An angiogenesis-associated gene-based signature predicting prognosis and immunotherapy efficacy of head and neck squamous cell carcinoma patients
Source: J Cancer Res Clin Oncol. 2024 Feb 12;150(2):91. doi: 10.1007/s00432-024-05606-8 (PMC10861726; doi:10.1007/s00432-024-05606-8)

APOH

Normal Tissue	Tumor Tissue

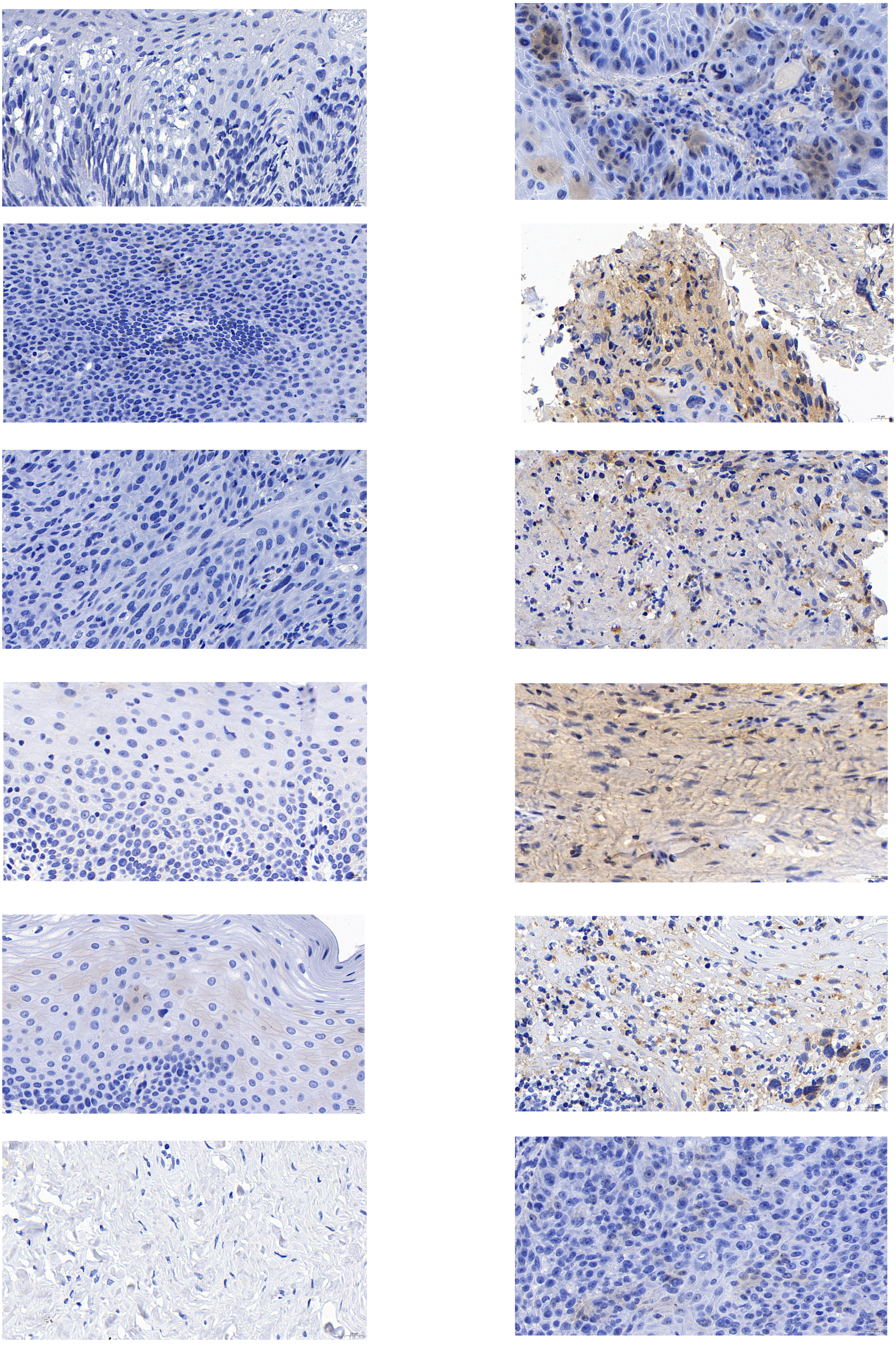

MSX1

Normal Tissue	Tumor Tissue

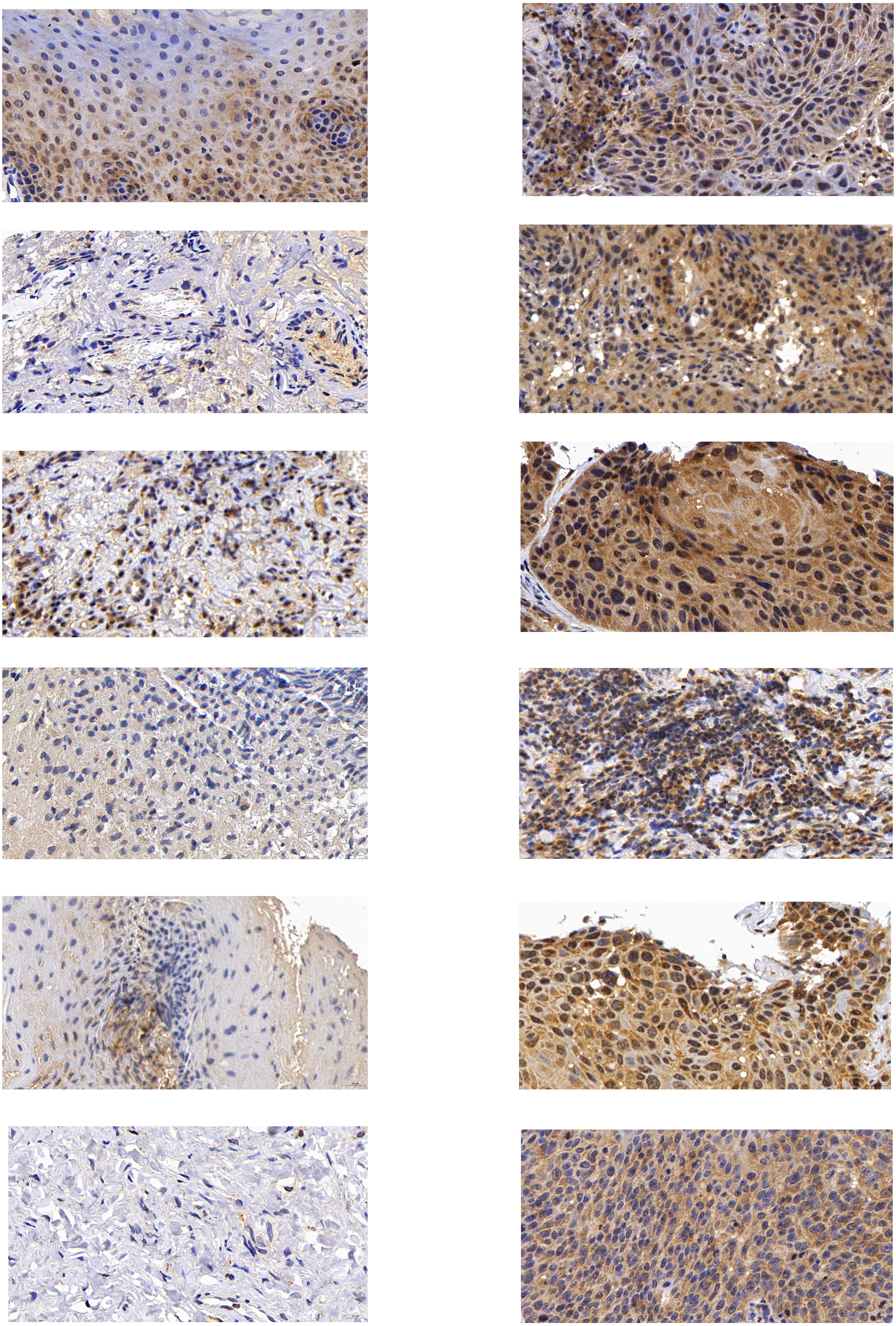

OLR1

Normal Tissue	Tumor Tissue

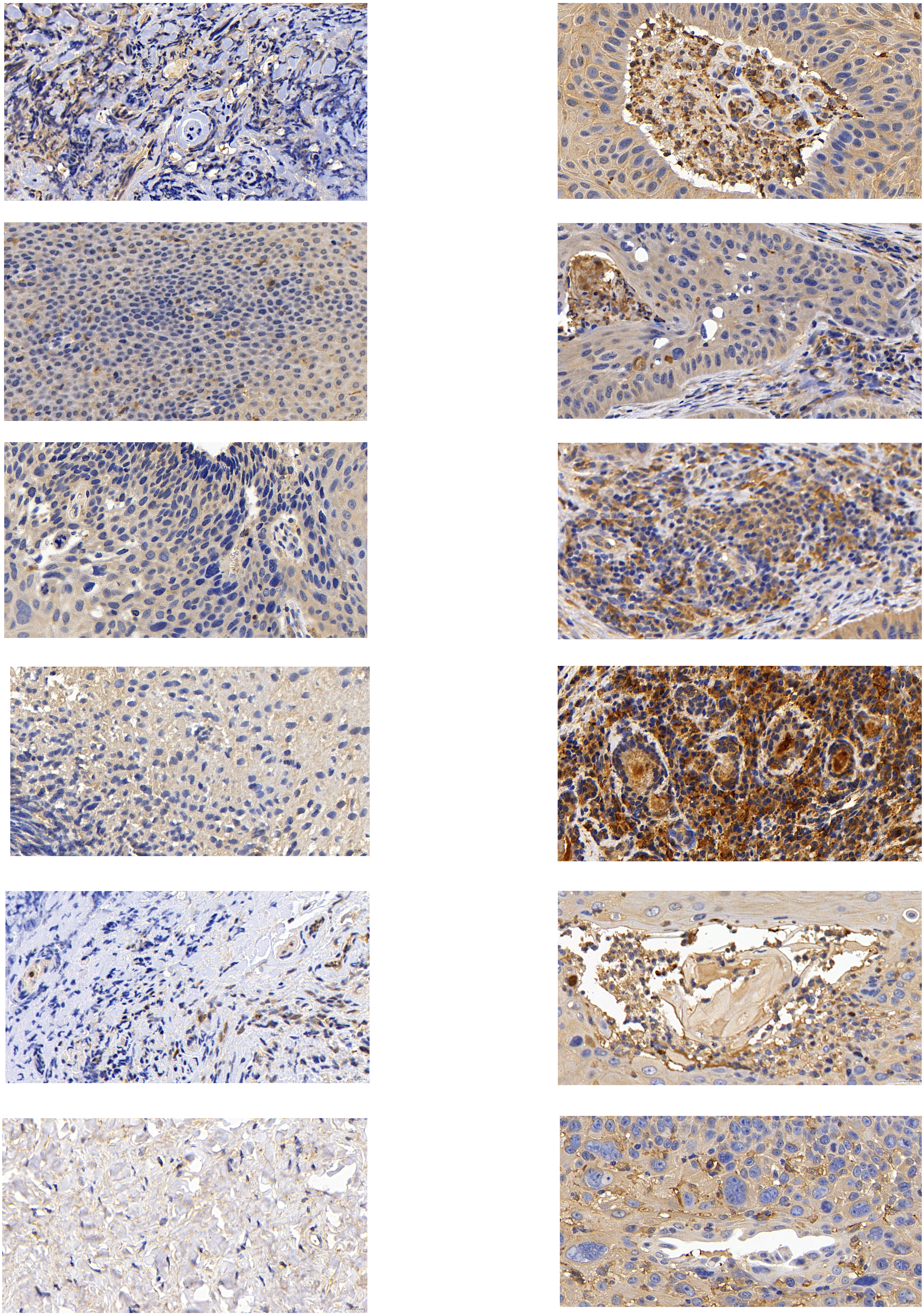

PDGFA

Normal Tissue | **Tumor Tissue**

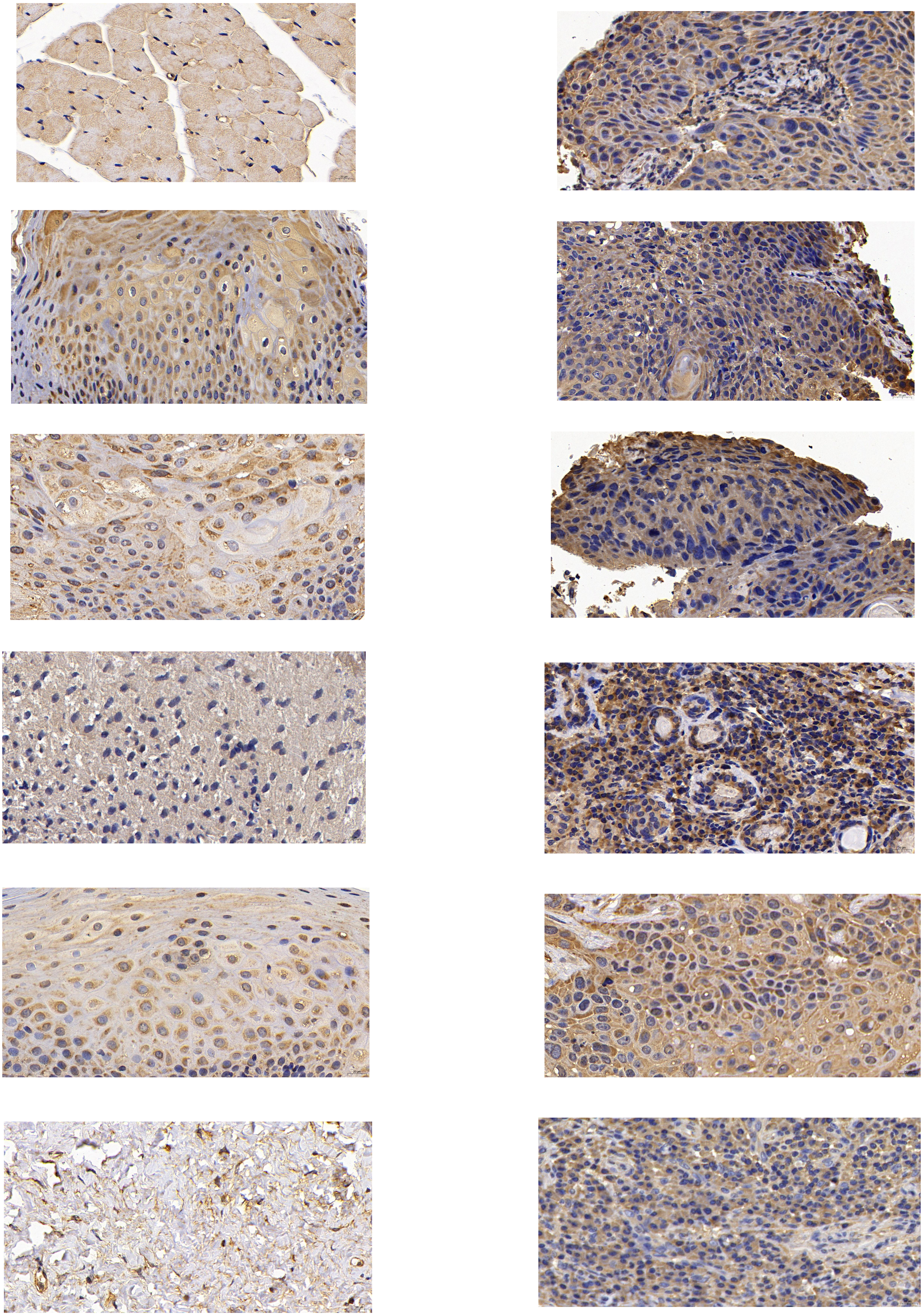

S100A4

Normal Tissue	Tumor Tissue

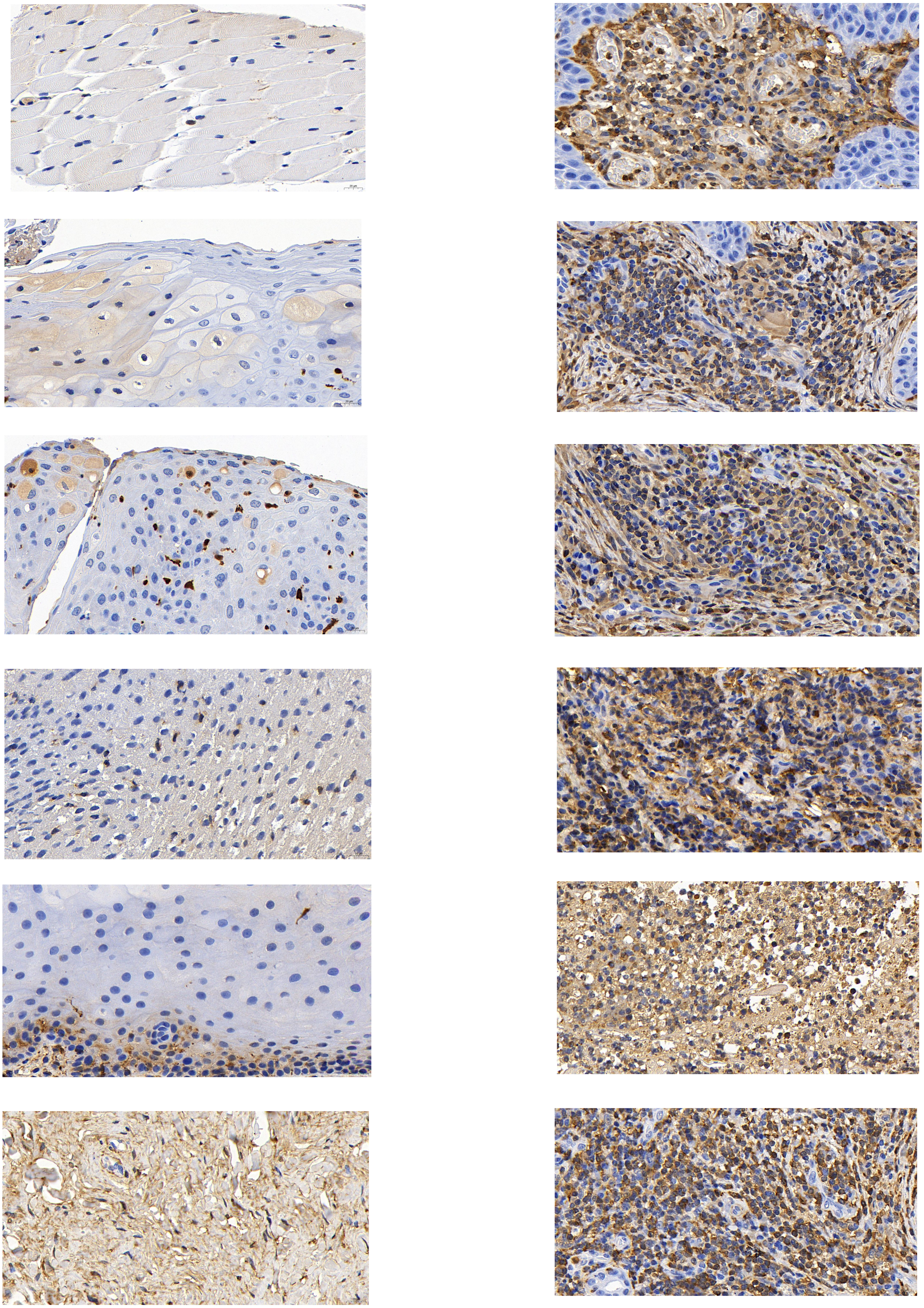

SERPINA5

Normal Tissue | Tumor Tissue

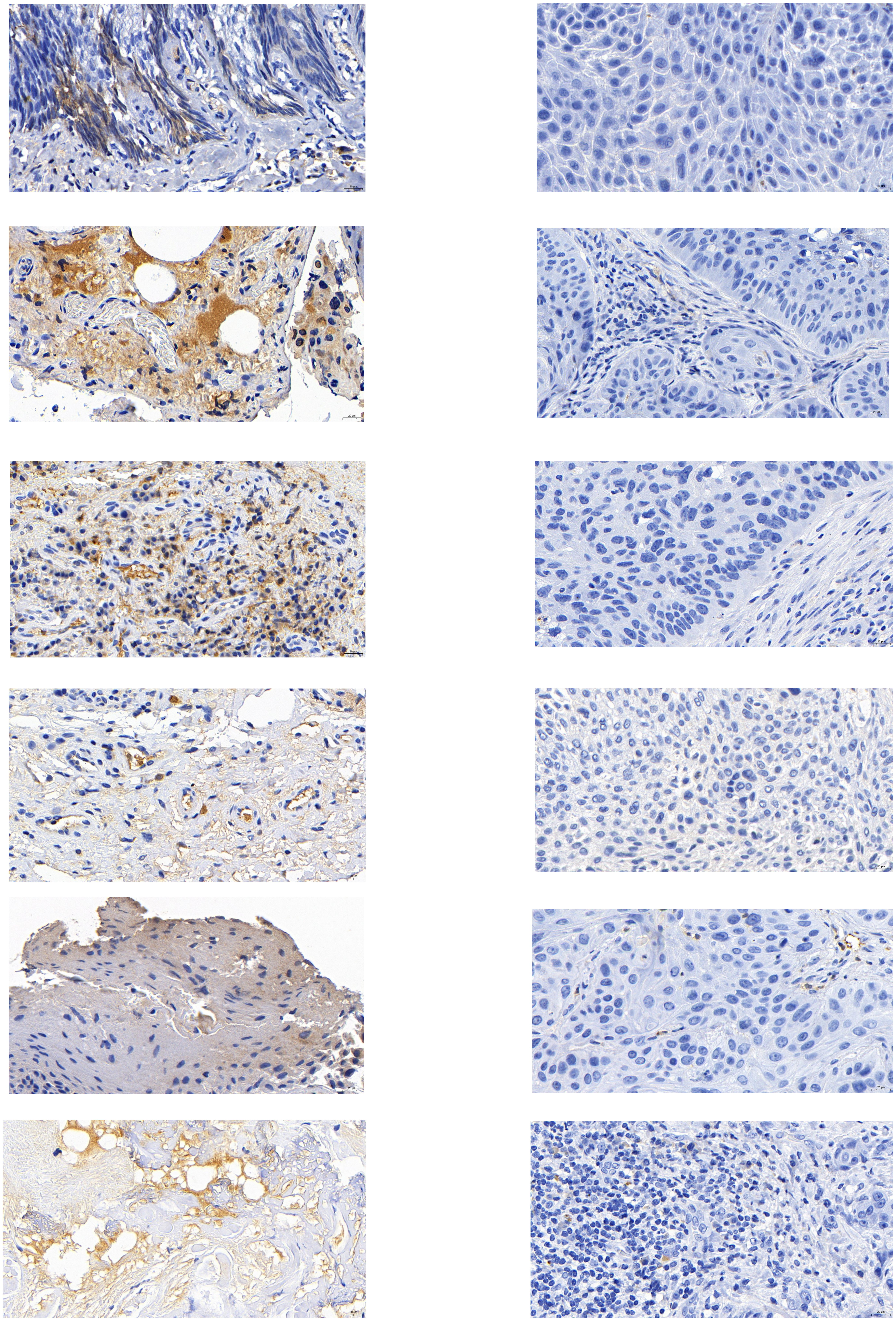

Supplement: Supplementary file 3 — Supplementary file 1 IHC staining results for all six pairs of tissues. Supplementary file3 (PDF 22085 KB) [file 432_2024_5606_MOESM3_ESM.pdf]
