# Supplementary figures and images for: An angiogenesis-associated gene-based signature predicting prognosis and immunotherapy efficacy of head and neck squamous cell carcinoma patients
Source: J Cancer Res Clin Oncol. 2024 Feb 12;150(2):91. doi: 10.1007/s00432-024-05606-8 (PMC10861726; doi:10.1007/s00432-024-05606-8)

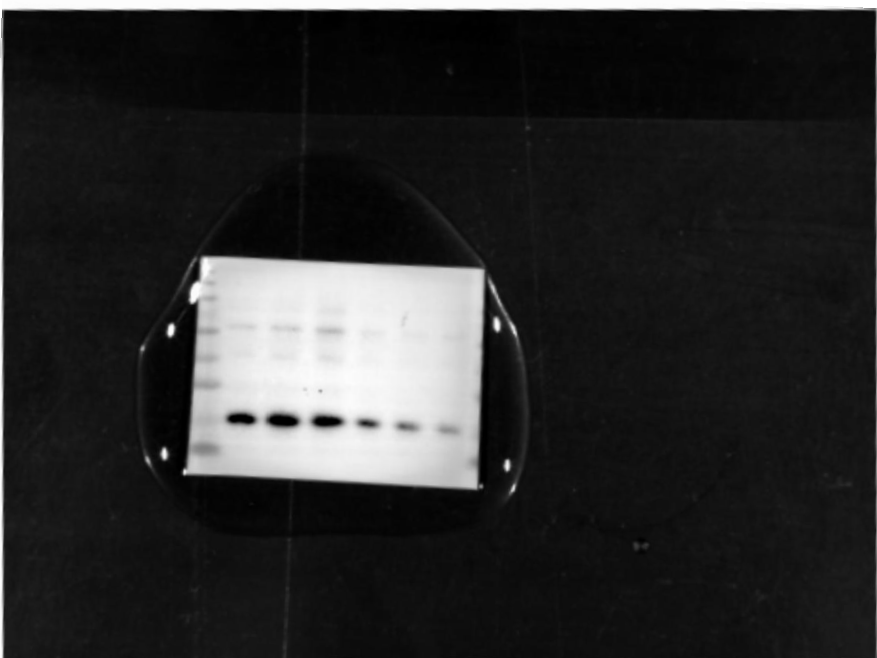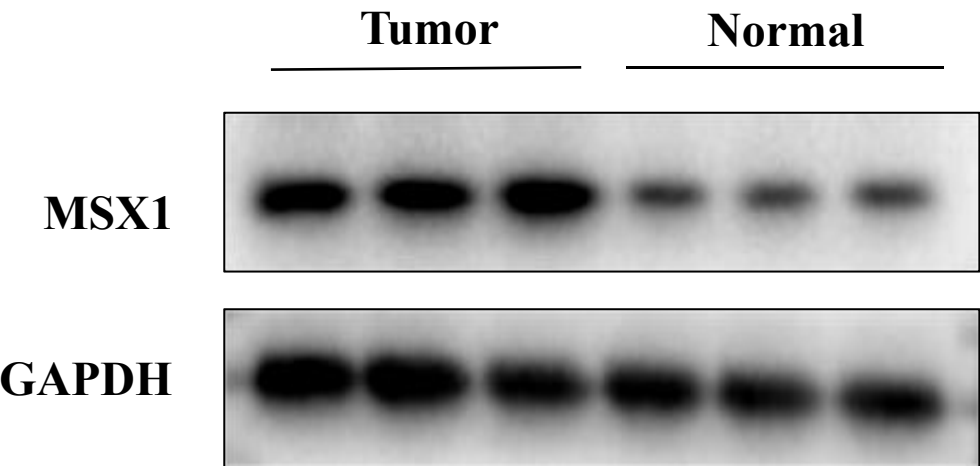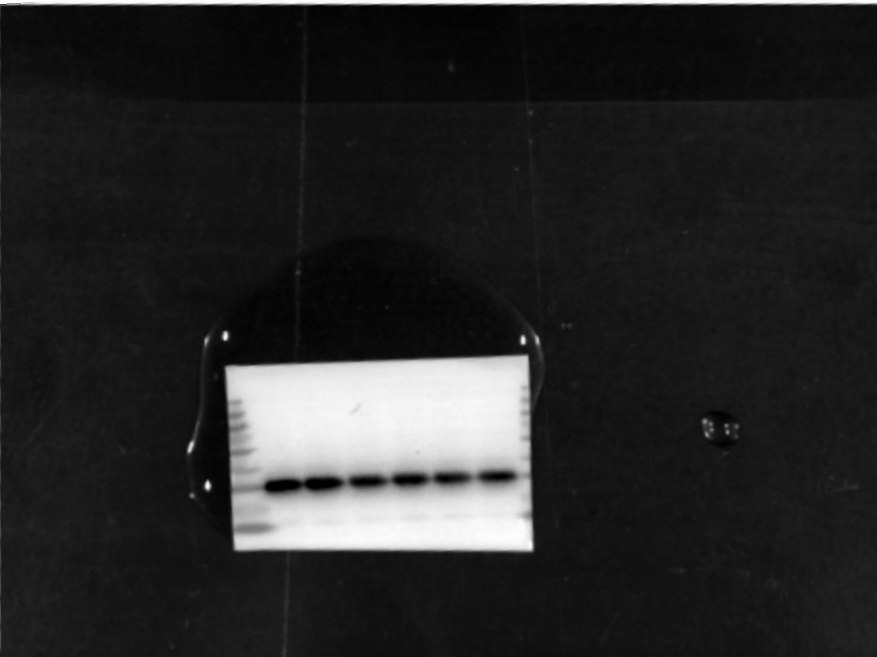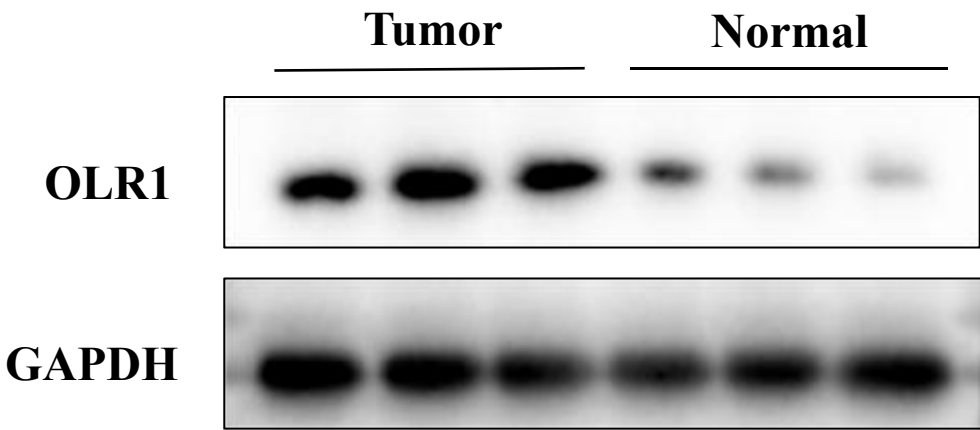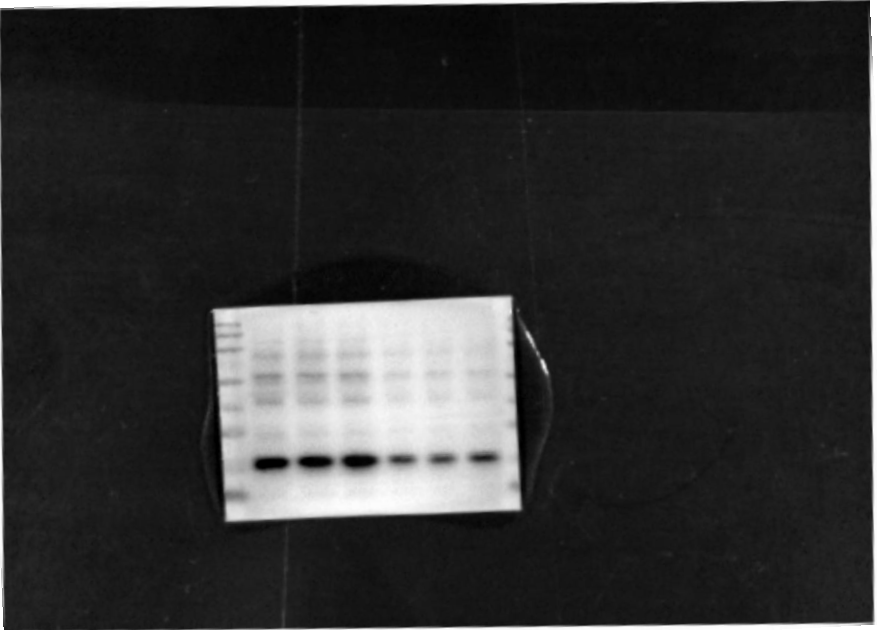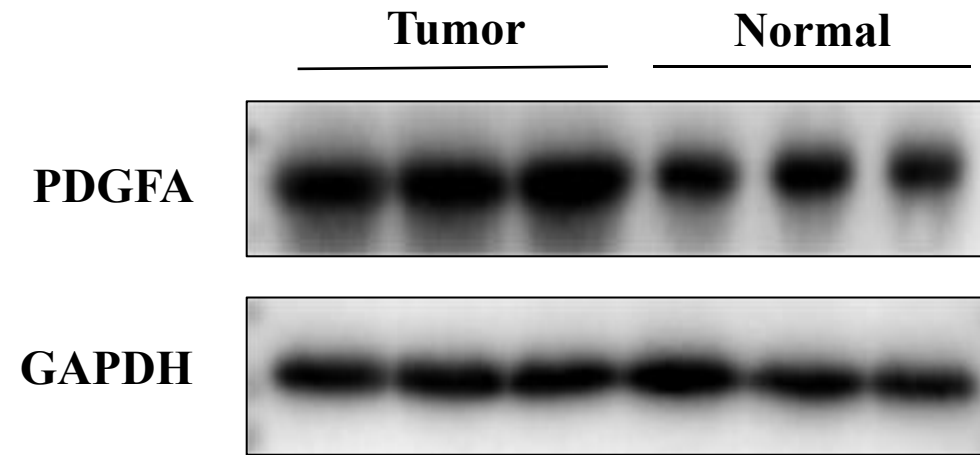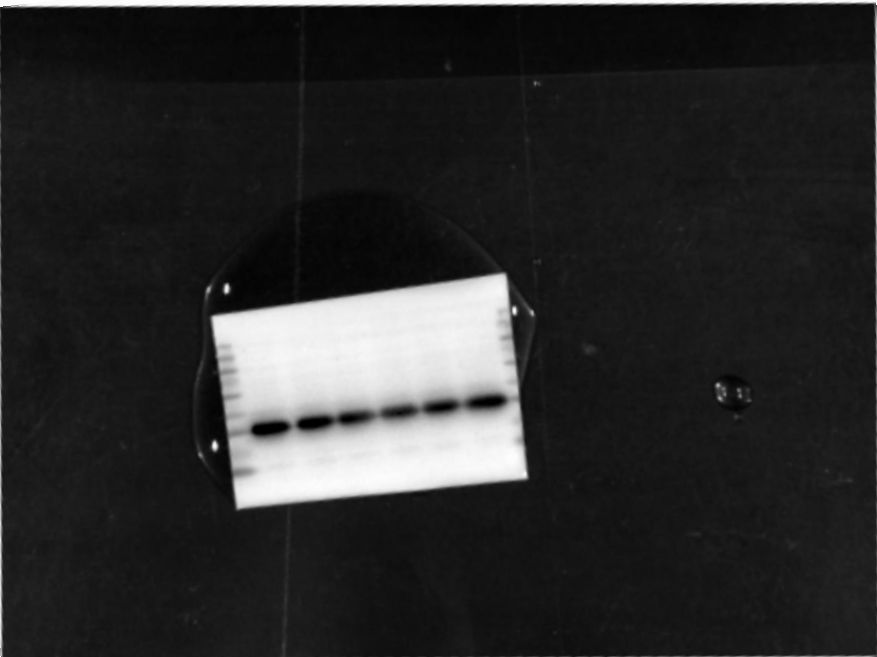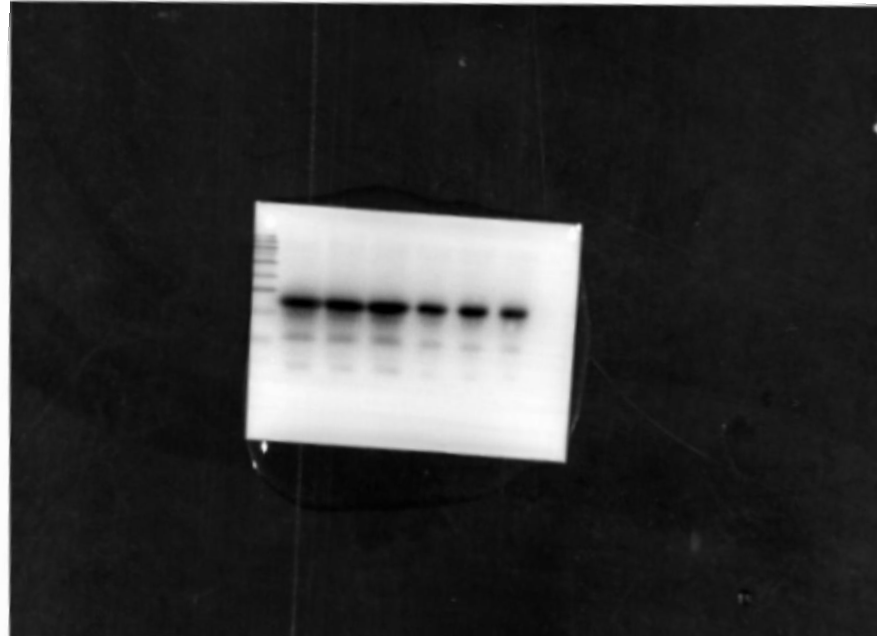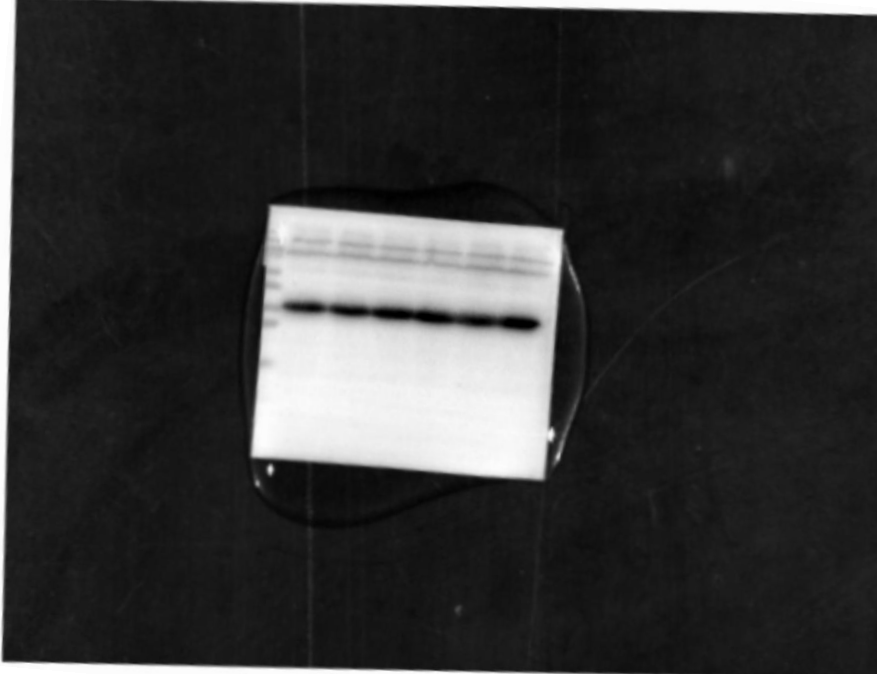

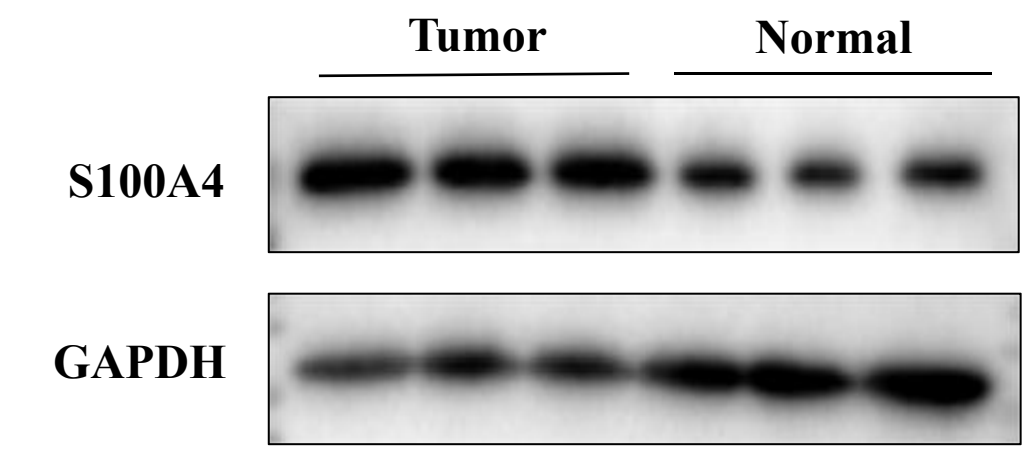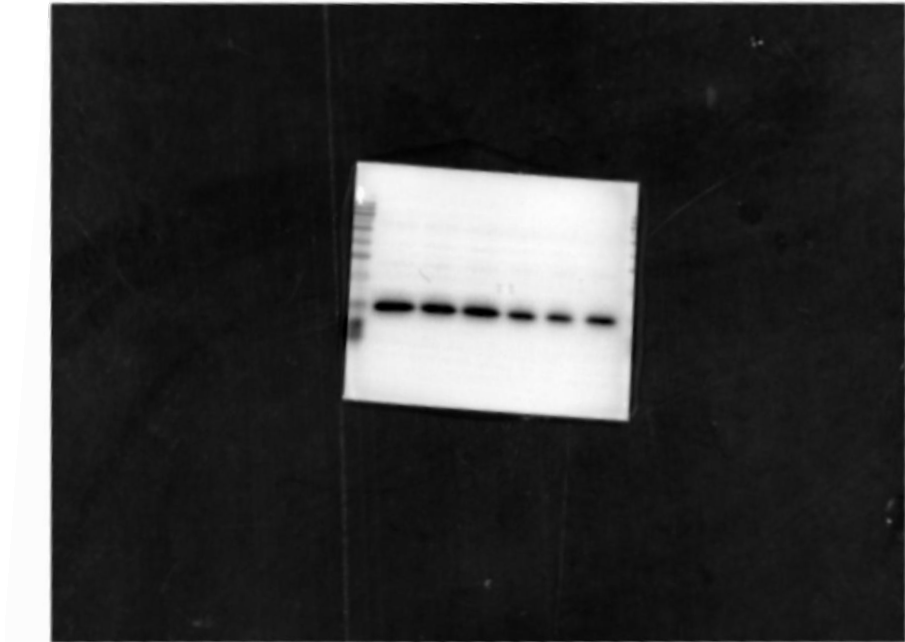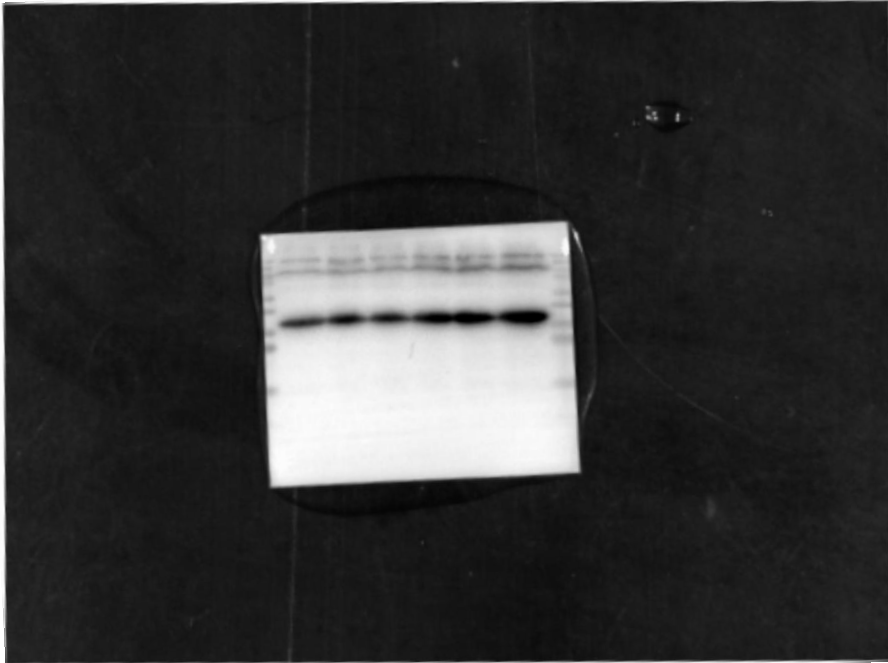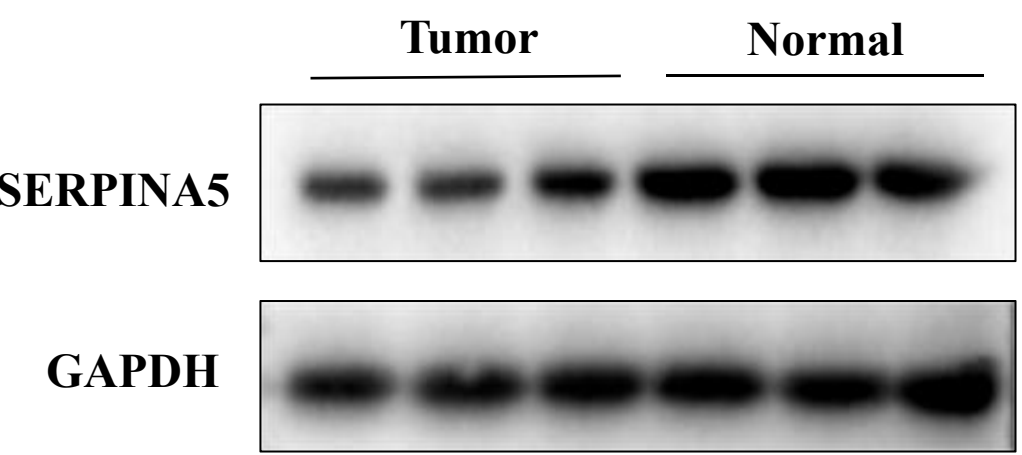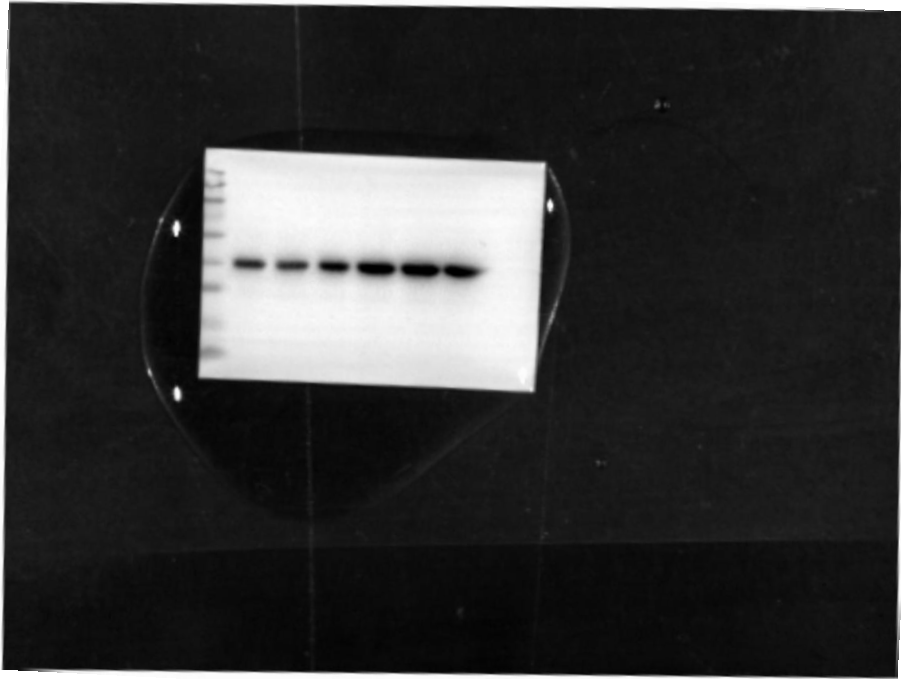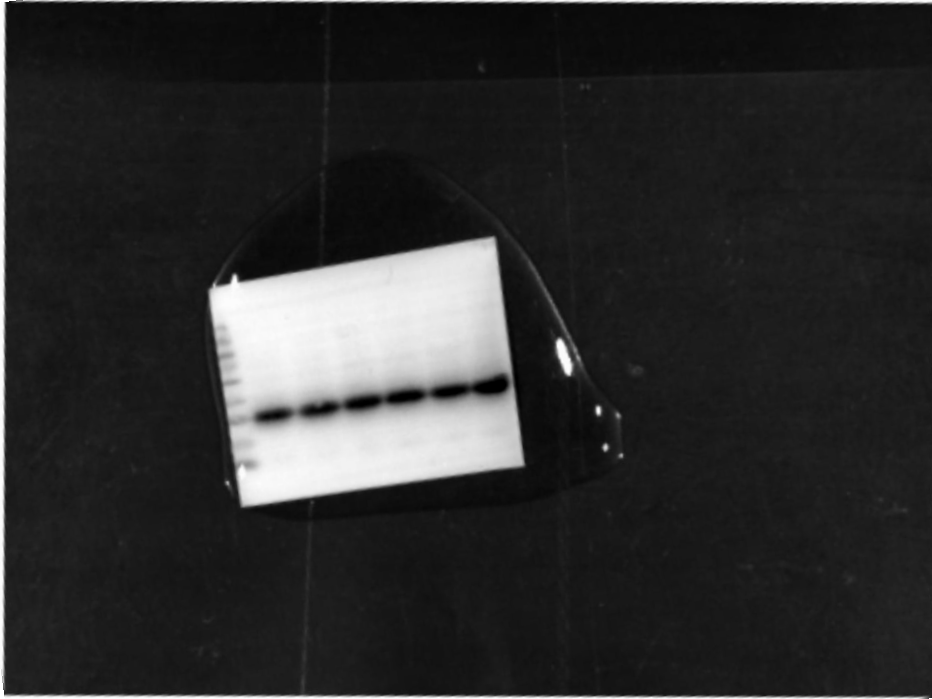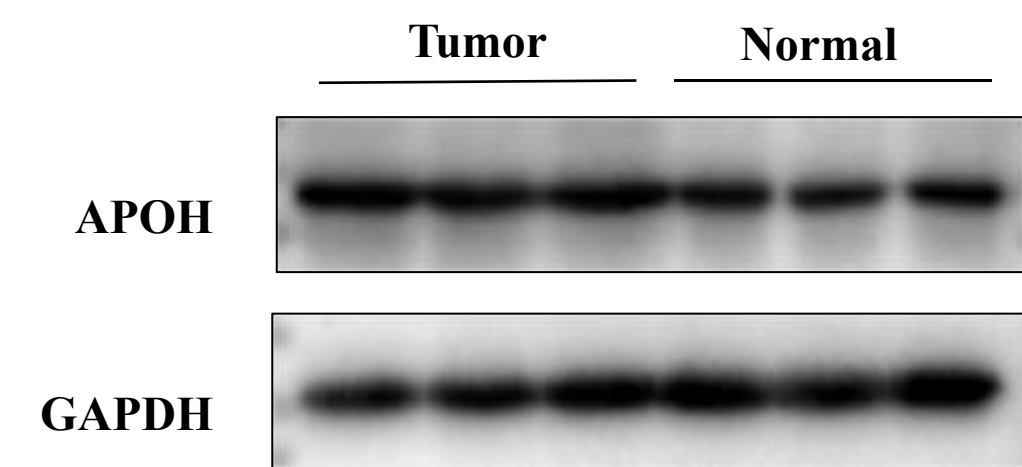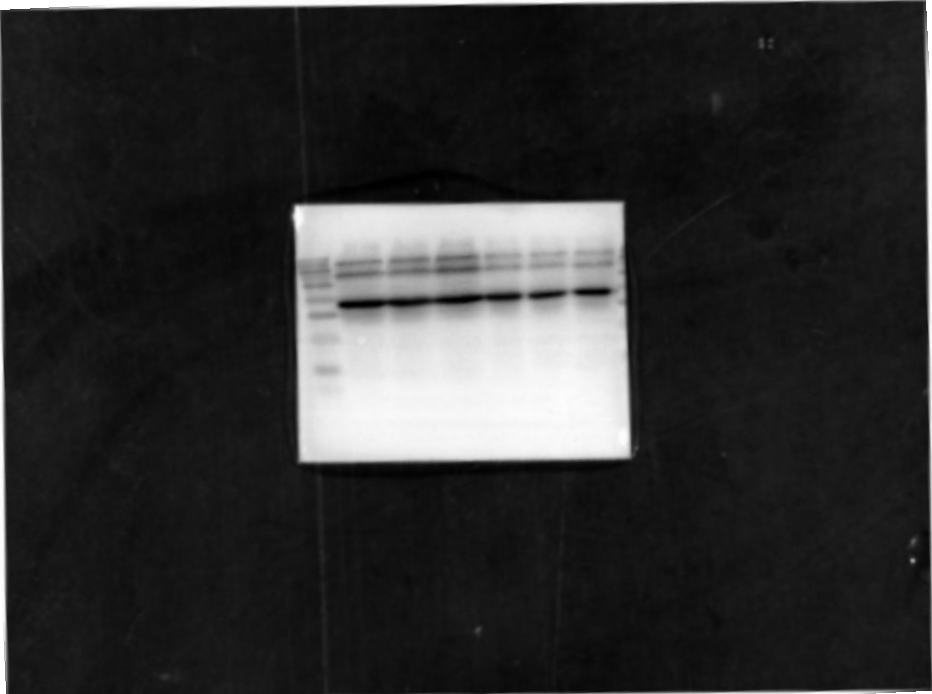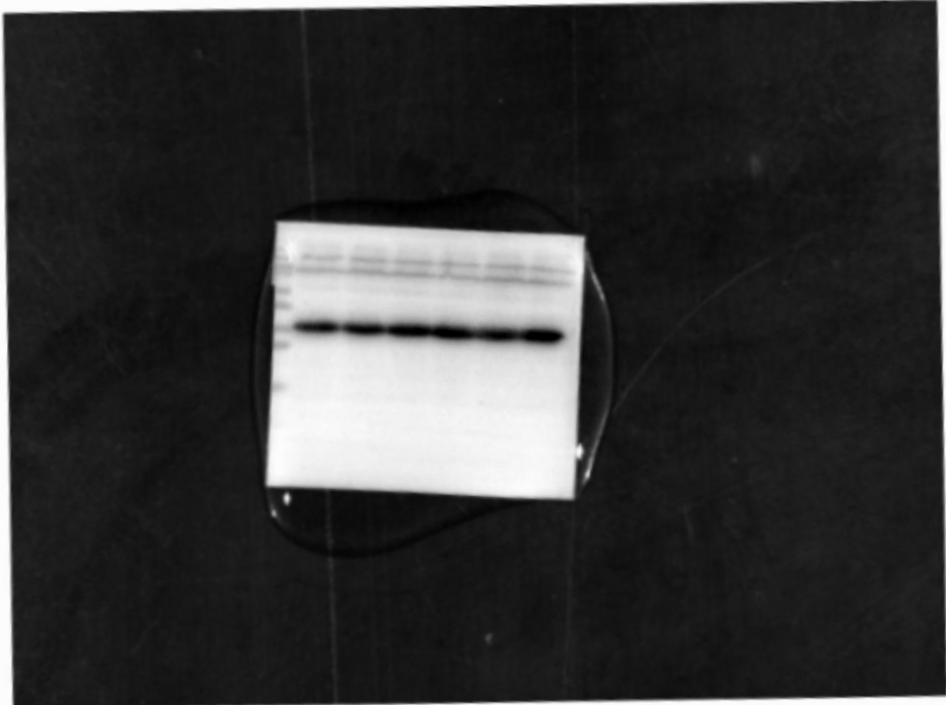

Supplement: Supplementary file 4 — Supplementary file 2 Original whole membrane of western blotting bands. Supplementary file4 (PDF 596 KB) [file 432_2024_5606_MOESM4_ESM.pdf]
